# Artificial intelligence in mental health care: a systematic review of diagnosis, monitoring, and intervention applications

**DOI:** 10.1017/S0033291724003295

**Published:** 2025-02-06

**Authors:** Pablo Cruz-Gonzalez, Aaron Wan-Jia He, Elly PoPo Lam, Ingrid Man Ching Ng, Mandy Wingman Li, Rangchun Hou, Jackie Ngai-Man Chan, Yuvraj Sahni, Nestor Vinas Guasch, Tiev Miller, Benson Wui-Man Lau, Dalinda Isabel Sánchez Vidaña

**Affiliations:** 1 Rehabilitation Research Institute of Singapore, Nanyang Technological University, Singapore, Singapore; 2 School of Public Health, LKS Faculty of Medicine, The University of Hong Kong, Hong Kong, Hong Kong; 3 Department of Rehabilitation Sciences, The Hong Kong Polytechnic University, Hong Kong, Hong Kong; 4 Department of Building Environment and Energy Engineering, The Hong Kong Polytechnic University, Hong Kong, Hong Kong; 5 Mental Health Research Center, The Hong Kong Polytechnic University, Hong Kong, Hong Kong

**Keywords:** artificial intelligence, chatbot, machine learning, mental health

## Abstract

Artificial intelligence (AI) has been recently applied to different mental health illnesses and healthcare domains. This systematic review presents the application of AI in mental health in the domains of diagnosis, monitoring, and intervention. A database search (CCTR, CINAHL, PsycINFO, PubMed, and Scopus) was conducted from inception to February 2024, and a total of 85 relevant studies were included according to preestablished inclusion criteria. The AI methods most frequently used were support vector machine and random forest for diagnosis, machine learning for monitoring, and AI chatbot for intervention. AI tools appeared to be accurate in detecting, classifying, and predicting the risk of mental health conditions as well as predicting treatment response and monitoring the ongoing prognosis of mental health disorders. Future directions should focus on developing more diverse and robust datasets and on enhancing the transparency and interpretability of AI models to improve clinical practice.

## Introduction

Artificial intelligence (AI) is defined as the ability of a system to interpret external data, learn from it, and accomplish specific goals through adaptation (Haenlein & Kaplan, 2019). AI, particularly machine learning, has shown promise in surpassing human capabilities in various tasks such as medical image analysis, clinical documentation, and patient monitoring (Bohr & Memarzadeh, 2020; Davenport & Kalakota, 2019). Machine learning is a technique that uses advanced statistical and probabilistic methods to build systems that improve through experience, enabling prediction and categorization of data, particularly in mental health research (Chung & Teo, 2022). Traditional machine learning is commonly used in precision medicine to predict successful treatments based on patient attributes and treatment context (Davenport & Kalakota, 2019). Neural networks are advanced algorithms in machine learning that are designed to mimic the human brain function, enabling them to solve complex problems like image and speech recognition (Chung & Teo, 2022). Neural networks are employed to categorize patients and determine the likelihood of developing specific diseases (Davenport & Kalakota, 2019). Deep learning is a subset of machine learning that utilizes neural networks to automatically learn and solve complex problems, including image and speech recognition, and natural language processing (Chung & Teo, 2022). Deep learning utilizes multiple layers of features to predict outcomes, such as disease prognosis and patient mortality in cancer cases (Lu et al., 2022; Zhang et al., 2022). Another application of deep learning is speech recognition through natural language processing, which aims to understand human language through speech recognition, text analysis, and translation (Locke et al., 2021; Nassif et al., 2019), for assisting in tasks such as creating, analyzing, and classifying clinical documentation, transcribing patient interactions, and conducting conversations (Buchlak et al., 2022; Casey et al., 2021; Davenport & Kalakota, 2019; Kreimeyer et al., 2017).

AI in the field of mental health has witnessed significant growth (Cecula et al., 2021) with studies exploring its potential in the early detection of mental illnesses, treatment planning (Ćosić et al., 2020; Johnson et al., 2021), speech signal analysis in therapy sessions (Goldberg et al., 2020), and continuous patient monitoring (Bohr & Memarzadeh, 2020). Given the rising global demand for accurate diagnosis, improved monitoring, and effective interventions in mental health, AI holds promise as a powerful tool. The demand for mental health diagnosis and treatment further intensified during the COVID-19 pandemic, with a notable increase in depressive symptoms, anxiety, and distress worldwide (Davenport et al., 2020; Latoo et al., 2021; Moreno et al., 2020). To address the substantial increase in global demand for mental health resources, the use of AI tools has emerged as a potential solution. By leveraging AI, various applications can be developed to support and enhance mental health services. AI-assisted diagnosis tools can enable early detection and treatment (Ćosić et al., 2020; Johnson et al., 2021). AI-powered monitoring can facilitate continuous and remote mental health assessments, reducing the need for patients to travel to healthcare facilities (Graham et al., 2019). AI-based interventions have the potential to address this demand by offering scalable and adaptable solutions to different populations (Bickman, 2020; Koutsouleris et al., 2022).

To advance AI technology in the field of mental health and overcome its current limitations, it is crucial to have a comprehensive understanding of how AI can be applied throughout the patient journey. The need for a comprehensive review of the application of AI in mental health research and clinical practice is underscored by the growing reliance on technology to address pressing mental health challenges. As AI systems become increasingly proficient in interpreting data and producing actionable insights, they present an opportunity to enhance traditional approaches to mental health diagnostics, monitoring, and interventions. The increasing demand for mental health services, exacerbated by the COVID-19 pandemic, emphasizes the importance of leveraging AI to facilitate early detection of mental illnesses, optimize treatment planning, and provide continuous patient support. By systematically evaluating the existing literature, this review will elucidate how AI can transform mental health care, potentially leading to more accurate diagnoses, personalized treatment plans, and efficient resource allocation, thereby contributing significantly to the overall understanding of AI’s role in strengthening mental health systems worldwide.

AI in mental health is hampered by difficulties in obtaining high-quality, representative data, along with data security concerns, lack of training resources, and fragmented formats (Koutsouleris et al., 2022). Additionally, the belief that clinical judgment outweighs quantitative measures slows advancements in digital health care and AI applications (Koutsouleris et al., 2022). This systematic review aims to analyze the current status of AI in mental health care focusing specifically on its application in the areas of diagnosis, monitoring, and intervention as well as to identify the limitations, challenges, and ethical considerations associated with the use of AI technologies. Focusing this systematic review on three critical domains – diagnosis, monitoring, and intervention – allows for a targeted analysis of the multifaceted ways in which AI can enhance mental health care. In the diagnosis domain, exploring AI’s role can reveal its potential for early identification of mental health conditions, improving patient outcomes through timely intervention. In terms of monitoring, AI technologies can enable ongoing assessments that are essential for tracking patient progress and adapting treatment plans effectively. Finally, examining AI-assisted interventions showcases how scalable digital solutions can address the growing demand for accessible mental health resources. By dissecting these three domains, the review will not only highlight the strengths and limitations of AI applications but also address ethical considerations, ultimately guiding future research and innovation in mental health technology. This systematic review was prepared to answer the following questions:

1. How is AI used in diagnosing mental health illnesses, monitoring disease progression and treatment effectiveness, and conducting AI-assisted mental health interventions?
2. What are the limitations, challenges, and ethical concerns in the application of AI technologies in mental health?

## Methods

### Search strategy

The systematic review was conducted following PRISMA guidelines, and it was registered on PROSPERO (registration number: CRD42023388503). The literature search was conducted using the Cumulative Index to Nursing and Allied Health (CINAHL), Cochrane Central Register of Controlled Trials (CCRT), PubMed, PsycINFO, and Scopus databases from inception to August 2024. The search terms and search strategy that were used to retrieve relevant research studies are shown in Table 1. Filters were applied to retrieve research studies, including “Trials” for CCRT; “Full text,” “English language,” and “Randomized controlled trials” for CINAHL; “Clinical trial” and “English” for PsycINFO; “Full text,” “Clinical trial,” “Randomized controlled trial,” and “English” for PubMed; and “Article,” “Journal,” “English,” and “Doc title, abstract, keyword” for Scopus.

**Table 1.** Search terms and database search strategy

ID	Search term
	Tool
1	Machine learning
2	Deep learning
3	Artificial intelligence
4	Artificial neural network
5	Machine intelligence
6	Computer reasoning
7	Computational intelligence
8	1 OR 2 OR 3 OR 4 OR 5 OR 6 OR 7
	Health domain
9	Mental health
10	Mood disorders
11	9 OR 10
	Approach
12	Effectiveness
13	Prediction
14	Diagnosis
15	Treatment
16	Intervention
17	Monitoring
18	12 OR 13 OR 14 OR 15 OR 16 OR 17
	Type of study
19	Randomized controlled trial
20	Clinical trial
21	19 OR 20
22	8 AND 11 AND 18 AND 21

### Inclusion and exclusion criteria for study selection

General, domain-specific, and outcome measures inclusion and exclusion criteria were set for study selection.

### General

Studies using AI-assisted diagnosis tools, AI-monitored treatment effectiveness and prognosis, or AI-based interventions in the context of mental health were included. Studies that did not include mental health outcomes or primarily targeted disorders such as dementia, attention-deficit/hyperactivity disorder, or autism spectrum disorders as well as drug abuse were excluded. Also, systematic reviews, meta-analyses, classical reviews, protocols, book chapters, conference presentations, and studies not written in English were excluded.

## Domain-specific

### Diagnosis

Studies that applied AI in detecting the presence of mental health disorders, predicting the risk of having mental health disorders, and identifying features that are associated with mental health disorders were included. Studies that classified subgroups of mental illnesses were excluded as diagnoses had already been made.

### Monitoring

Those studies were included that adopted AI either to collect data for monitoring and predicting the ongoing prognosis of a mental health disorder or to monitor treatment effects. Studies that used AI to predict treatment-related mental health improvement or the risk of symptom remission prior to treatment initiation were excluded, as this review aimed to focus on monitoring treatment effectiveness and treatment-related mental health prognosis in clinical practice.

### Intervention

Studies that applied any form of AI-assisted interventions were included. Studies that did not use AI-assisted interventions or used AI in other aspects of the research, such as data analysis and outcome prediction were excluded.

### Outcome measures

The findings were presented in a systematic and narrative form, including the AI approaches used in mental health, the domain of mental health care, in which AI was applied, the presence and severity of mental health disorders or symptoms, and the accuracy or effectiveness of the AI-based tool. The application, limitations, challenges, and ethical concerns of AI in mental health were also critically discussed.

### Selection of relevant studies

Two authors independently conducted the database search and the selection of studies. The study selection was carried out according to the inclusion and exclusion criteria. After the article search and removing duplicates, the titles and abstracts of the retrieved research studies were screened. The next screening for study selection was conducted by revising the full text. After selecting the studies, the authors reviewed the list of studies included. Discrepancies were resolved by a third author.

### Data extraction

Three authors were involved in the data extraction, that is, one author per domain and one additional author revised the extracted data and resolved any discrepancies. The data extracted included AI approaches used in mental health, the mental health care domain in which AI was applied, the AI tool, sample size, effectiveness, as well as limitations, challenges, and ethical considerations of AI in mental health. Study investigators were contacted regarding any missing data.

### Quality assessment

The National Heart, Lung, and Blood Institute’s (NHLBI) quality assessment tools were used to examine the quality of the studies included. The studies encompassed various types, including controlled intervention studies, observational cohort and cross-sectional studies, case-control studies, and before-after (pre–post) studies without a control group, with assessments conducted using different NHLBI tools. The number of items assessed for each study type was 14, 14, 12, and 12, respectively. For the scoring method, each item was categorized as “yes,” “no,” or “other” (e.g., “cannot determine,” “not applicable,” or “not reported”). The overall quality score, categorized as “good,” “fair,” or “poor,” was not simply a cumulative total, but rather a qualitative assessment derived from response patterns. Reviewers considered essential factors that could influence the validity of the study (https://www.nhlbi.nih.gov/health-topics/study-quality-assessment-tools). Two independent authors performed the quality appraisal, with a third author helping to resolve any disagreements.

## Results

**Figure 1.**
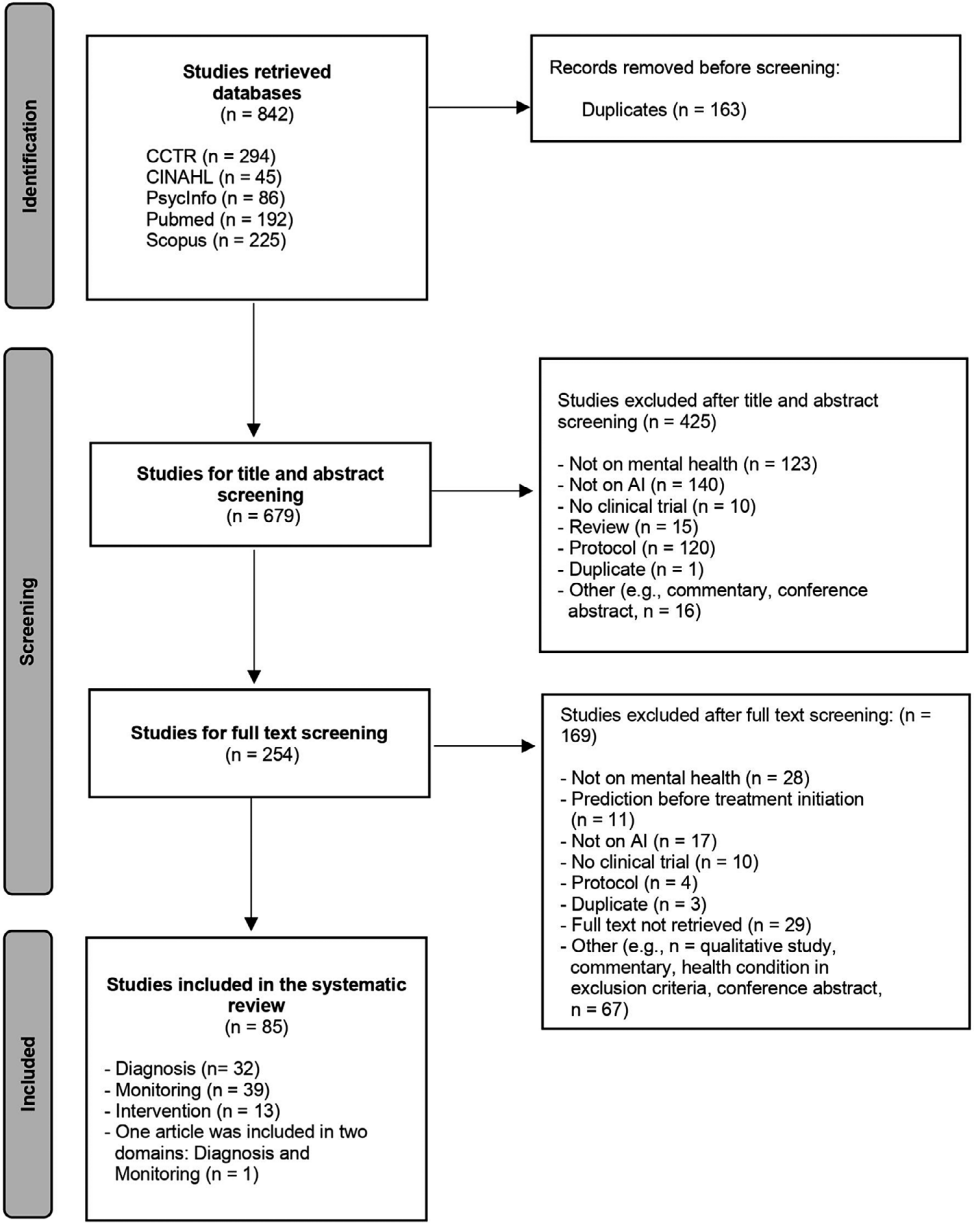
PRISMA flowchart of study identification, screening, and selection.

A total of 842 research studies were retrieved from five databases, including CINAHL, CCRT, PubMed, PsycINFO, and Scopus. After screening and removing duplicates, a total of 32 studies were included in diagnosis, 39 in monitoring, 13 in intervention, and one in both diagnosis and monitoring (Figure 1).

### Diagnosis

Thirty-two studies in the diagnosis domain trained and developed machine learning algorithms to detect and predict mental health conditions (Table 2). The target population (*n* = 327,625) involved individuals who developed or were susceptible to developing mental health conditions. The most common algorithms included support vector machine, a supervised learning algorithm for classification and regression tasks that finds the optimal hyperplane to maximize the margin between different classes (Adler et al., 2022; Byun et al., 2019; Chilla et al., 2022; Das & Naskar, 2024; Ebdrup et al., 2019; Geng et al., 2023; Marquand, Mourão-Miranda, Brammer, Cleare, & Fu, 2008; Matsuo et al., 2022; Mohamed et al., 2023; Mongan et al., 2021; Pestian et al., 2016; Schnack et al., 2014; Setoyama et al., 2016; Susai et al., 2022; Tate et al., 2020) and random forest, an ensemble learning method that improves predictive accuracy by aggregating the outputs of multiple decision trees, reduces overfitting while enhancing model robustness (Andersson et al., 2021; Chen et al., 2024; Chilla et al., 2022; Ebdrup et al., 2019; Hüfner et al., 2022; Jacobson et al., 2022; Kourou et al., 2023; Lønfeldt et al., 2023; Manikis et al., 2023; Matsuo et al., 2022; Mohamed et al., 2023; Setoyama et al., 2016; Tate et al., 2020). Machine learning was used to diagnose specific mental disorders such as depression (Byun et al., 2019; Carrillo et al., 2018; Chen et al., 2024; Das & Naskar, 2024; Du et al., 2021; Geng et al., 2023; Hüfner et al., 2022; Maglanoc et al., 2020; Marquand et al., 2008; Setoyama et al., 2016; Xu et al., 2018), schizophrenia (Chilla et al., 2022; Ebdrup et al., 2019; Liang et al., 2018; Schnack et al., 2014), and suicide (Cook et al., 2016; Jacobson et al., 2022; Jankowsky et al., 2024; Lyu & Zhang, 2019; Pestian et al., 2016; Simon et al., 2019; Tsui et al., 2021; Yang et al., 2024); and it was less frequently applied in the diagnoses of anxiety (Hüfner et al., 2022; Maglanoc et al., 2020), bipolar disorder (Schnack et al., 2014), obsessive-compulsive disorder (Lønfeldt et al., 2023), and postpartum depression (Andersson et al., 2021; Matsuo et al., 2022); and to detect mental health symptoms such as depressive, anxious, and schizophrenia symptoms (Adler et al., 2022; C Manikis et al., 2023; Kourou et al., 2023; Maekawa et al., 2024; Mongan et al., 2021; Susai et al., 2022; Tate et al., 2020) as well as outcomes associated with quality of life (Hüfner et al., 2022; Manikis et al., 2023; Xu et al., 2018). The predictors that were commonly used to detect and predict mental health conditions included demographic information, socioeconomic data, clinical history, physiological data, psychometric data, medical scan biomarkers, and semantic contents (Table 2). Demographic information, socioeconomic data, and clinical history were retrieved from electronic health records. Examples of medical scans used as input in the AI models were MRI scans (Chilla et al., 2022; Ebdrup et al., 2019; Marquand et al., 2008), EEG signals (Du et al., 2021), and HRV signals (Geng et al., 2023), whereas biomarkers included aqueous metabolites in blood plasma (Setoyama et al., 2016), gray matter density (Maglanoc et al., 2020; Schnack et al., 2014), and proteomics data from plasma samples (Mongan et al., 2021).

**Table 2.** Studies on AI-assisted diagnosis in mental health

Ref.	Subject description	Mental health condition	Aim	AI-based method	Models	Variables	Predictors	Results and accuracy	Conclusions	
Chen et al. (2024)	Patients with MDD and healthy controls (*n* = 156)	MDD	To detect lifetime diagnosis of MDD and nonremission status	Machine learning models combined with natural language processing	RF Logistic regression SVC K-Nearest neighbors DT Naive bayes Artificial neural networks	Clinically psychiatric diagnosis and HAMD–17 (cutoff score of 7)	Subjective happiness level Actigraphy (physical activity and sleep estimation) Facial expression (inner brow raising, brow lowering, cheek raising, lip corner pulling, lip corner depressing) Voice (articulation rate, pause duration, pause variability, pause rate) Self-reference and negative emotion HADS-D	Artificial neural networks (use all variables as predictors to predict patients with MDD and HAMD–17 > 7) Sensitivity: 0.64 Specificity: 0.96 PPV: 0.78 NPV: 0.91 Artificial neural networks (not include HADS-Depression as predictors to predict patients with MDD) Sensitivity: 0.86 Specificity: 0.74 PPV: 0.76 NPV: 0.85 Naive bayes (not include HADS-D as predictors to predict patients with MDD) Sensitivity: 0.90 Specificity: 0.71 PPV: 0.74 NPV: 0.88	The prediction performance of artificial neural networks was generally more favorable compared to other machine learning methods for both lifetime MDD diagnosis and nonremission (HAMD–17 > 7) with the fusion of all digital variables	
Das and Naskar (2024)	Patients with depression and controls (DAIC-WOZ dataset, *n* = 219; MODMA dataset, *n* = 52)	Depression	To identify symptoms of depression among individuals from their speech and responses	Machine learning models	e.g., SVM, CNN, LSTM, DT	PHQ–8 binary and professional judgment	Variables from an audio spectrogram	DAIC-WOZ dataset Accuracy: 90.26% MODMA dataset Accuracy: 90.47%	A novel deep learning-based approach using audio signals for automatic depression recognition has demonstrated superior detection accuracy compared to existing methods	
Maekawa et al. (2024)	Individuals with depressive symptoms and healthy controls (*n* = 35628)	Depressive symptoms	To identify individuals with depressive symptoms	Machine learning algorithms	Stochastic gradient descent (evaluated by two different methods of feature selection: Bayesian network or Markov blanket)	PHQ–9	Age, education, gender, income Postural balance problems, shortness of breath, how old people feel they are, the ability to do usual activities, chest pain, chronic back problems, sleep problems, verbal abuse	Bayesian network: AUC are 0.736, 0.801, 0.809 in three different datasets (use different variables as predictors)	Bayesian network feature selection method outperformed Markov blanket selection method The models have emphasized the importance of the ability to do usual activities, chest pain, and sleep problems as key indicators for detecting depressive symptoms	
Yang et al. (2024)	Suicidal ideators and suicide attempters (*n* = 438)	Suicide attempts	To identify predictors for suicide attempts and suicides	Machine learning	Logistic regression Penalized regression (elastic net regression)	The number of suicide attempts reported	136 variables in total Sociodemographic characteristics (age, sex, living status, employment status, religion) Clinical information (medical and psychiatric illness, treatment, and previous suicidal thoughts and attempts) Psychopathological evaluation (PHQ–9, BAI, AUDIT, BIS–11, ETI-SF, SRS, SQ for KNHANES-SF, C-SSRS)	Classical logistic regression (136 variables included): AUC: 0.535 Elastic net regression (136 variables included): AUC: 0.812 Classical logistic regression (15 variables included): AUC: 0.926 Accuracy: 91.2% Elastic net regression (15 variables included): AUC: 0.912 Accuracy: 90.0%	Young age, suicidal ideation, previous suicidal attempts, anxiety, alcohol abuse, stress, and impulsivity were significant predictors of suicide attempts	
C Manikis et al. (2023)	Women with highly treatable breast cancer (*n* = 706)	Depression, anxiety, overall mental health, and QoL	To identify women at risk of poor mental health, declining mental health and declining global QoL following a diagnosis of breast cancer	Machine learning algorithms	Balanced RF	HADS–14 European Organisaztion for Research and Treatment of Cancer Quality of life Questionnaire-Cancer	Sociodemographics: six variables (month 0), two variables (month 3) Lifestyle: four variables (month 0) Medical: 11 variables Breast cancer and treatment-related: 17 variables Psychosocial characteristics: seven domains	Model A for patients with poor mental health at month 0 Model B for patients with good mental health at month 0 Model C for patients with good QoL at month 0 i: use variables at month 0 and month 3 as predictors ii: use clinical and biological variables at month 0 with other variables at month 6 as predictors 12-month AUC Model Ai: 0.81 Model Aii: 0.78 Model Bi: 0.86 Model Bii: 0.79 Model Ci: 0.77 Model Cii: 0.79	The top predictors of adverse mental health and QoL outcomes include common variables in clusters: negative affect, cancer coping responses/self-efficacy to cancer, sense of control/optimism, social support, lifestyle factors, and treatment-related symptoms	
Geng et al. (2023)	Patients with MDD and healthy controls (*n* = 80)	MDD	To optimize initial screening for MDD in both male and female patients	Machine learning algorithms	SVM ERTC	PHQ–9	24 HRV-related variables (analyzed by 5-min short-term electrocardiogram signals during nighttime sleep stages): time domain and frequency domain Gender	SVM: AUC: 0.853 Accuracy: 79.29% ERTC: AUC: 0.945 Accuracy: 86.32%	Through feature importance analysis, we found that MeanNN, MedianNN, pNN20, and gender were the most important features HRV parameters during sleep stages can be used for the identification of MDD patients	
Kourou et al. (2023)	Women diagnosed with stage I–III breast cancer with a curative treatment intention (*n* = 600)	Symptoms of anxiety and depression	To predict adverse mental health outcomes among patients who manifest fairly good initial emotional response to the diagnosis and the prospect of cancer treatments	Adaptive machine learning algorithms	Balanced RF	HADS–14	Sociodemographic, lifestyle, medical variables, and self-reported psychological characteristics were recorded at diagnosis and assessed 3 months after diagnosis	Model 1: use all variables at month 0 and month 3 as predictors Model 2: not include mental health and subjective QoL ratings at months 0 and 3 12-month AUC Model 1: 0.864 Model 2: 0.790	The top predictors of adverse mental health and QoL outcomes include common variables in clusters: negative affect, cancer coping responses/self-efficacy to cancer, sense of control/optimism, social support, lifestyle factors, and treatment-related symptoms	
Lønfeldt et al. (2023)	Adolescents with mild-to-moderate-severe obsessive-compulsive disorders (*n* = 9)	Obsessive-compulsive disorders	To detect obsessive-compulsive disorders episodes in the daily lives of adolescents	Machine learning models	Logistic regression RF Feedforward neural networks Mixed-effect RF	Obsessive-compulsive disorders events marked by participants	Blood volume pulse, external skin temperature, and electrodermal activity and heart rate (calculated by blood volume pulse)	10-fold random cross-validation Average accuracy: >70% Recall: 50% Precision: 66% Average AUC: 0.8	Better performance was obtained when generalizing across time compared to across patients Generalized temporal models trained on multiple patients outperformed personalized single-patient models RF and mixed-effect RF models consistently achieved superior accuracy, reaching 70% accuracy in random and participant cross-validation	
Adler et al. (2022)	Patients with schizophrenia, schizoaffective disorder, or psychosis non-specified in treatment, and university students (*n* = 109)	Mental health symptoms	To explore if machine learning models can be trained and validated across multiple mobile sensing longitudinal studies (CrossCheck and StudentLife) to predict mental health symptoms	Machine learning algorithms	GBRT SVM	EMA	Mobile sensing data of sleep quality and stress	Improved model performance for predicting sleep: CrossCheck (W = 53,200, p = 0.007, RBC = 0.14) StudentLife (W = 63,089, p < 0.001, RBC = 0.35) Improved model performance for predicting stress: CrossCheck (W = 55,373, p < 0.001, RBC = 0.18)	Machine learning models trained across longitudinal mobile sensing study datasets generalized and provided a more efficient method to build predictive models of adding what they predicted, e.g., sleep and stress	
Chilla et al. (2022)	Patients with schizophrenia and healthy controls (*n* = 234)	Schizophrenia	To classify schizophrenia and healthy control cohorts using a diverse set of neuroanatomical measures	Machine learning	k-Nearest Neighbors Logistic regression SVC Linear SVC Nu-SVC Decision trees RF	A structured clinical interview for DSM-IV Disorders-Patient Version Clinical history, existing medical records, and interviews with significant others (e.g., family members, spouse, children)	MRI imaging on subcortical volumes, cortical volumes, cortical areas, cortical thickness & mean cortical curvature	Classification performance was comparable between independent measure sets, with accuracy, sensitivity, and specificity, ranging 70%–73%, 73%–81%, and 57%–61%, respectively Employing a diverse set of measures (measures were merged and used in Ensemble) resulted in improved accuracy, sensitivity, and specificity, with ranges of 77%–87%, 79%–98%, and 65%–74%, respectively	Subcortical and cortical measures and Ensemble methods achieved better classification performance on people with schizophrenia	
Hüfner et al. (2022)	Individuals resided in Austria aged ≥16 or resided in Italy aged ≥18 who were confirmed with SARS-CoV–2 infection and were not under hospitalization (*n* = 2,050)	Depression, anxiety, overall mental health and QoL	To identify indicators for poor mental health following COVID–19 outpatient management and to identify high-risk individuals Machine learning algorithm	Machine learning algorithm	RF	PHQ– 4 Self-perceived Overall Mental Health and QoL rated with 4-point Likert scale	201 surveyed demographic, socioeconomic, medical history, COVID–19 course, and recovery parameters	RMSE of Austria data: 0.15–0.18 and Italy data: 0.21–0.23	Machine learning achieved moderate-to-good performance in mental health risk prediction	
Jacobson et al. (2022) Note: Also included in the monitoring domain.	Users who made queries related to mental health screening tools to the Microsoft Bing search engine between December 1, 2018, and January 31, 2020 (*n* = 126,060)	Suicidal ideation, active suicidal intent	To examine the impact and qualities of widely used, freely available online mental health screening on potential benefits, including suicidal ideation and active suicidal intent	Machine learning algorithm	RF	Suicidal ideation and suicidal intent search queries identified by seed keywords Rating of common queries by two independent raters Filtering of a multi- variate regression model	Exposure to online screening tools and past search behaviors	AUC of: Suicidal ideation: 0.58 Suicidal intent: 0.60	Websites with referrals to in-person treatments could put persons at greater risk of active suicidal intent. Machine learning’s prediction accuracy of suicidal ideation and intent was moderate	
Matsuo et al. (2022)	Pregnant women who delivered at ≥35 weeks of gestation (*n* = 34,710)	PPD	To develop and validate machine learning models for the prediction of postpartum depression and to compare the predictive accuracy of the machine learning models with conventional logistic regression models	Four machine learning algorithms	Conventional logistic regression models Ridge regression Elastic net Kernel-based SVM RF	EPDS	Maternal baseline (18 variables) Pregnancy-related (four variables) Delivery-related (eight variables) Neonatal (eight variables) Postpartum at two-week postpartum checkup (three variables)	AUC assessing the predictive accuracy: Model 1 (using variables collected in the first to second trimester): Logistic (0.634) Ridge regression (0.638) Elastic net (0.637) Kernel-based SVM (0.530) RF (0.629) Model 2 (using variables collected before discharge from hospitals): Logistic (0.626) Ridge regression (0.630) Elastic net (0.628) Kernel-based SVM (0.569) Random Forest (0.613) Model 3 (using all variables, including the two-week postpartum checkup): Logistic (0.697) Ridge regression (0.702) Elastic net (0.701) Kernel-based SVM (0.642) RF (0.688)	The approach used did not achieve better predictive performance than the conventional logistic regression models	
Susai et al. (2022)	Participants from NEURAPRO, aged between 13 and 40 who fulfilled one of the criteria for at-risk state defined by CAARMS (*n* = 158)	Psychosis: functioning	To investigate the combined predictive ability of blood-based biological markers on functional outcome	Machine learning model	SVM	SOFAS	Clinical predictors: four demographic variables including sex, age, smoking status, BMI, and seven symptom scale scores Biomarker predictors: ten cytokines; 157 proteomic markers; and ten fatty acid markers	Model based on clinical predictors: Accuracy: 56.4 AUC: 0.63 Model based on biomarker predictors: Accuracy: 58.9 AUC: 0.62 Model based on clinical and biomarker predictors: Accuracy: 57.5 AUC: 0.58	Machine learning model based on clinical and biological data poorly predicted functional outcome in clinical high-risk participants	
Andersson et al. (2021)	Pregnant women who were 18 years of age or older (*n* = 4,313)	PPD	To predict women at risk for depressive symptoms at six weeks postpartum, from clinical, demographic, and psychometric questionnaire data available after childbirth Machine learning algorithm	Machine learning algorithm	Ridge Regression LASSO Regression Gradient Boosting Machines DRF XRT Naive Bayes Stacked Ensembles models	EPDS	BP Psychometric data from RS, SOC, and VPSQ	Accuracy based on BP dataset: Ridge Regression: 70% LASSO Regression: 71% DRF: 70% XRT: 72% Gradient Boosting Machines: 70% Stacked Ensembles models: 70% Naive Bayes: 70% Accuracy based on combined dataset (BP + RS, SOC, and VPSQ): Ridge Regression: 67% LASSO Regression: 70% DRF: 71% XRT: 73% Gradient Boosting Machines: 68% Stacked Ensembles models: 65% Naive Bayes: 69%	All machine learning models had similar performance based solely on BP dataset; there were greater variations in model performance for the combined dataset	
Du et al. (2021)	College students (*n* = 30)	Depression	To design a deep learning-based mental health monitoring scheme to detect depression in college students	Deep learning	Convolutional neural network model	Confirmation with diagnosis of depression based on questionnaires and the bodily feelings	EEG signal	The model showed a classification accuracy score of 97.54%	The proposed deep learning-based mental health monitoring scheme achieved a high accuracy rate in detection of depression using EEG data	
Mongan et al. (2021)	EU-GEI participants who met clinical high-risk criteria of psychosis at baseline ALSPAC participants who did not report psychotic experiences at age 12 (*n* = 344)	Psychosis	To investigate whether proteomic biomarkers may aid prediction of: Transition on to psychotic disorder in people at high-clinical risk of psychosis Adolescent psychotic experiences in the general population	Machine learning algorithms	SVM	For the transition to psychotic disorders in the clinical high-risk: CAARMS interview Contact with the clinical team or review of clinical records For the adolescent psychotic experiences in the general population: Psychosis-Like Symptoms Interview at age 18	Proteomic data from plasma samples	For the transition to psychotic disorders in the clinical high-risk: Model based on clinical and proteomic data: AUC: 0.95 PPV: 75.0% NPV: 98.6% Model based on clinical data: AUC: 0.48 PPV: 37.1% NPV: 63.4% Model based on clinical and proteomic data: AUC: 0.96 PPV: 79.0% NPV: 100% For the adolescent psychotic experiences in the general population: AUC: 0.74 PPV: 67.8% NPV: 75.8%	Models based on proteomic data demonstrated excellent predictive performance for the transition to psychotic disorder in clinically high-risk individuals. Models based on proteomic data at age 12 had fair predictive performance for psychotic experiences at age 18	
Tsui et al. (2021)	Inpatients and emergency department patients aged 10–75 (*n* = 45,238)	First-time suicide attempt	To predict first-time suicide attempts from unstructured (narrative) clinical notes and structured EHR	NLP	Naive Bayes LASSO regression RF EXGB	ICD–9 and ICD–10	Unstructured data (clinical notes): history and physical examination, progress, and discharge summary notes. Structured data: demographics, diagnosis, healthcare utilization data, and medications	AUC of prediction window smaller or equal to 30 days: Full-feature (involved both structured and unstructured data) EXGB: 0.932 Structured-feature only EXGB: 0.901 Full-feature LASSO: 0.909 Full-feature LASSO: 0.884 Full-feature Naive Bayes: 0.766 Full-feature Random Forest: 0.900	Using both structured and unstructured data resulted in significantly higher accuracy than structured data alone	
Maglanoc et al. (2020)	Depression patients from outpatient clinics and healthy controls (*n* = 241)	Depression, Anxiety	To classify patients and controls, and to predict symptoms for depression and anxiety	Machine learning	Shrinkage discriminant analysis	M.I.N.I. Becks Depression Inventory Becks Anxiety Inventory	Brain components, including cortical macrostructure (thickness, area, gray matter density), white matter diffusion properties, radial diffusivity and resting-state functional magnetic resonance imaging (fMRI) default mode network amplitude Sex Age	Classifying patients and controls: AUC: 0.6194 Accuracy (proportion of correct classification): 0.6169 Sensitivity (ability to correctly detect cases): 0.6991 Specificity (ability to correctly detect controls): 0.4292 Predicting depression symptoms: RMSE: 10.72 Predicting anxiety symptoms: RMSE: 8.181 Predicting age: RMSE: 6.764	Machine learning revealed low model performance for discriminating patients from controls and predicting symptoms for depression and anxiety, but had high accuracy for age	
Tate et al. (2020)	Twins born between 1994 and 1999 (*n* = 7,638)	Mental health problems: parent-rated emotional symptoms, conduct problems, prosocial behavior, hyper- activity/ inattention, and peer relationship problems	To investigate if various machine learning techniques outperform logistic regression in predicting mental health problems in mid-adolescence.	Machine learning algorithms	RF XGBoost Logistic regression Neural network SVM	Strengths and Difficulties Questionnaire	Birth information, physical illness, mental health symptoms, environmental factors such as neighborhood and parental income	AUC and 95% interval of: Logistic Regression: 0.700 (0.665–0.734) Compared to: XGBoost: 0.692 (0.660–0.723) Random Forest: 0.739 (0.708–0.769) SVM: 0.736 (0.707–0.765) Neural Network: 0.705 (0.671–0.737)	All models performed with relatively similar accuracy; machine learning algorithms were no more significant statistically than logistic regression	
Byun et al. (2019)	MDD patients and healthy controls who were matched for age and gender (*n* = 78)	MDD	To investigate the feasibility of automated MDD detection based on heart rate variability features	Machine learning algorithm	SVM-RFE for feature selection SVM for classification	HAMD–17	Heart rate variability features extracted from electrocardiogram recordings	The best AUC of heart rate variability features selection for: SVM-RFE (based on two features): 0.742 Statistical filter (based on 5 features): 0.734 The highest accuracy of SVM classifier achieved based on: SVM-RFE (based on two features): 74.4 Statistical filter (based on five features): 73.1	SVM-RFE marginally outperformed the statistical filter with fewer number of heart rate variability features required in MDD classification	
Ebdrup et al. (2019)	Antipsychotic-naive first-episode schizophrenia patients and healthy controls (*n* = 104)	Schizophrenia, schizoaffective psychosis	To investigate whether machine learning algorithms on multimodal data can serve as a framework for clinically translating into diagnostic utility	Machine learning algorithms	Naive Bayes Logistic regression SVM Decision tree RF Auto- sklearn	A structured diagnostic interview to ensure fulfillment of ICD–10 diagnostic criteria of schizophrenia or schizoaffective psychosis	Four modalities Neurocognitive: DART, WAIS III, BACS, CANTAB Electrophysiology: CPTB Neuroanatomy: MRI scans Diffusion tensor imaging	Unimodal diagnostic accuracy: Diagnostic accuracy of cognition ranged between 60% and 69% Diagnostic accuracy for electrophysiology, sMRI and DTI ranged between 49% and 56%, and it did not exceed chance accuracy: ‘chance accuracy’ = 56% [(58/(46 patients +58 healthy controls) × 100%)] Multimodal diagnostic accuracy: None of the multimodal analyses with cognition plus any combination of one or more of the remaining modalities (electrophysiology, sMRI, and DTI) showed a significantly higher accuracy than cognition alone: the accuracy ranged between 51% and 68%	Only cognitive data, but no other modality, significantly discriminated patients from healthy controls No enhanced accuracies were noted by combining cognition with other modalities	
Jaroszewski et al. (2019)	Koko app users who signed up for the service (*n* = 39,450)	Mental health crisis: suicide (ideation, plan, and attempt), self-harm, eating disorder, physical abuse, unspecified abuse, emotional abuse and otherwise unspecified	To develop and evaluate a brief, automated risk assessment and intervention platform designed to increase the use of crisis resources among individuals routed to a digital mental health app who were identified as likely experiencing a mental health crisis	Machine learning classifiers	Recurrent neural networks with word embeddings	A binary classification of “crisis” or “not crisis, “crisis” defined as possibly at risk of serious, imminent physical harm, either through self-inflicted actions or through abuse from a third party	Semantic content of posts in real time	Performance: AUC: 0.93 Sensitivity: 0.64 Specificity: 0.98 PPV: 0.90 NPV: 0.93 Accuracy: 0.93	The classifiers demonstrated excellent performance in classifying risk of crisis from real time post, regardless of whether these posts were referring to the writer himself or a third party	
Lyu and Zhang (2019)	Suicide attempters randomly recruited through the hospital emergency and patient registration system (*n* = 659)	Suicide attempt	To establish the prediction model based on the Back Propagation neural network to improve prediction accuracy	Artificial Neural Network	Back Propagation Neural Network	Taken suicide attempt or not	Demographic information (such as age, gender, education level, marital status), family history of suicide, mental problem, aspiration strain, health status variables, hopelessness level, impulsivity, anxiety, depression, suicide attitude, negative life events, social support, coping skills, community environment etc.	The Back Propagation neural network: Sensitivity: 67.6% Specificity: 93.9% Total coincidence rate: 84.6% Traditional statistical methods such as multivariate Logistic regression: Sensitivity: 80.2% Specificity: 83.8% Total coincidence rate: 82.2%	Back Propagation neural network prediction model was superior in predicting suicide attempt	
Simon et al. (2019)	Members of the seven health systems, who had outpatient visits, either to a specialty mental health provider or a general medical provider when a mental health diagnosis was recorded (*n* = 25,373)	Suicide death Probable suicide attempt	To evaluate how availability of different types of health records data affect the accuracy of machine learning models predicting suicidal behavior Machine learning models	Machine learning models	Logistic regression with penalized LASSO variable selection	ICD–9th Revision cause of injury code indicating intentional self-harm (E950–E958) or undetermined intent (E980–E989) ICD–10th Revision diagnosis of self- inflicted injury (X60–X84) or injury or poisoning with undetermined intent (Y10–Y34)	Historical insurance claims data Sociodemographic characteristics (race, ethnicity, and neighborhood characteristics) Past patient-reported outcome questionnaires from electronic health records Data (diagnoses and questionnaires) recorded during medical visit	Prediction of suicide attempt following mental health visits: AUC of model 1 (limited to data typically available to an insurer or health plan): 0.843 AUC of model 4 (reflecting data that might inform predictions in an EHR environment capable of real-time calculation or updating risk scores): 0.850 Prediction of suicide death following mental health visits: AUC of model 1: 0.836 AUC of model 4: 0.861 Prediction of suicide attempt following general medical visits: AUC of model 1: 0.836 AUC of model 4: 0.853 Prediction of suicide death following general medical visits: AUC of model 1: 0.819 AUC of model 4: 0.833	For prediction of suicide attempt following mental health visits, model limited to historical insurance claims data performed approximately as well as model using all available data For prediction of suicide attempt following general medical visits, addition of data recorded during visits yielded improvement in model accuracy	
Carrillo et al. (2018)	Patients with treatment-resistant depression (*n* = 35)	Depression	To classify patients with depression and healthy control with machine learning algorithm	Natural speech algorithm combined with machine learning	Gaussian Naive Bayes classifier	Quick Inventory of Depressive Symptoms	AMT structured interview in which participants were asked to provide specific autobiographical memories in response to specific cue words	Mean accuracy of identifying patients with depression from controls was 82.85%	The natural speech analysis identified depression from the healthy control	
Liang et al. (2018)	First-episode patients with schizophrenia, MDD, and demographically matched healthy controls (*n* = 577)	Schizophrenia MDD	To investigate the accuracy of neurocognitive graphs in classifying individuals with first-episode schizophrenia and MDD in comparison with healthy controls	Machine learning algorithm	Graphical LASSO logistic regression	Wechsler Adult Intelligence Scale-Revised in China The computerized CANTAB Trail Making Test, parts A and B- Modified	Neurocognitive graphs based on cognitive features including general intelligence, immediate and delayed logical memory, processing speed, visual memory, planning, shifting, and psychosocial functioning	Classification accuracy of: First-episode schizophrenia and Healthy control: 73.41% MDD and Healthy control: 67.07% First-episode schizophrenia and MDD: 59.48%	Machine learning algorithm achieved moderate accuracy in classifying first-episode schizophrenia and MDD against healthy controls. Classification accuracy between first-episode schizophrenia and MDD was substantially lower	
Xu et al. (2018)	Postmenopausal obese or overweight, early-stage breast cancer survivors participating in a weight loss treatment (*n* = 333)	Depression and QOLm	To elicit bio-behavioral pathways implicated in obesity and health in breast cancer survivorship	Machine learning	Bayesian networks	SF–36 for QOLm Variable(s) for depression was not mentioned	Demographics and lifestyle Clinical factors Cancer treatment Coping Neighborhood Health Health behaviors	Insomnia predict depression with Strength = 0.93 Direction = 0.60 SE = 1.000 (0.257) Depression predict QOLm with Strength = 0.95 Direction = 0.97 SE = −7.029 (1.717) Sleep impairment predict QOLm with strength = 1.00 direction = 0.95 SE = −1.060 (0.094)	Higher level of insomnia is associated with higher level of depression Poor depression and sleep were associated with poorer QOLm	
Cook et al. (2016)	Adults discharged after self-harm from emergency services or after a short hospitalization (*n* = 1,453)	Suicidal Ideation, Heightened Psychiatric Symptoms	Developing and employing a predictive algorithm in a free- text platform (i.e., physician notes in EHRs, texts, and social media) to predict suicidal ideation and heightened psychiatric symptoms	Machine learning algorithm	NLP	Suicidal ideation by the question: “Have you felt that you do not have the will to live?” Heightened psychiatric symptoms measured by GHQ–12	Structured items (e.g., relating to sleep and well-being) Responses to one unstructured question, “how do you feel today?”	Suicidal ideation: NLP-based models using unstructured question: PPV: 0.61, Sensitivity: 0.56, Specificity: 0.57 Logistic regression prediction models using structured data: PPV: 0.73, Sensitivity: 0.76, Specificity: 0.62 Heightened psychiatric symptoms: NLP-based models using unstructured question: PPV: 0.56, Sensitivity: 0.59, Specificity: 0.60 Logistic regression prediction models using structured data: PPV: 0.79, Sensitivity: 0.79, Specificity: 0.85	NLP-based models were able to generate relatively high predictive values based solely on responses to a simple general mood question	
Pestian et al. (2016)	Suicidal (intervention group) or orthopedic (control group) teenager patients aged 13 to 17 admitted at the emergency department (*n* = 61)	Suicidal ideation	To evaluate whether machine learning methods discriminate between conversations of suicidal and non-suicidal individuals	NLP	SVM	C-SSRS SIQ UQ	Language	96.67% accurately matched the gold standard C-SSRS	Machine learning methods accurately distinguished between suicidal and non-suicidal teenagers	
Setoyama et al. (2016)	Patients with any depressive symptoms (HAMD–17 > 0), including both medicated and medication free (*n* = 115)	Depression and suicidal ideation	To create a more objective system evaluating the severity of depression, especially suicidal ideation	Machine learning	Partial least squares regression model Logistic regression Support vector machine Random Forest	HAMD–17 PHQ–9 Structured interview using M.I.N.I.	Aqueous metabolites in blood plasma	Each model on evaluating severity of depression showed a fairly good correlation with either value R2 = 0.24 (PHQ–9) and R2 = 0.263 (HAMD–17) The three models discriminated depressive patients with or without SI showed true rate > 0.7	Plasma metabolome analysis is a useful tool to evaluate the severity of depression An algorithm to estimate a grade of SI using only a few metabolites was successfully created	
Schnack et al. (2014)	Schizophrenia patients, bipolar disorder patients, and healthy controls selected from database (*n* = 334)	Schizophrenia and bipolar disorder	To classify patients with schizophrenia, bipolar disorder, and healthy controls on the basis of their structural MRI scans	Machine learning algorithms	Three SVM: M(sz-hc) to separate schizophrenia from healthy controls M(bp-sz) to separate bipolar from schizophrenia M(bp-hc) to separate bipolar from healthy controls	DSM-IV criteria for schizophrenia DSM-IV criteria for bipolar disorder	Gray matter density	M(sz-hc): Average accuracy rate 90.1% 92.4% of schizophrenia and 87.9% of healthy controls correctly classified M(bp-sz): Average accuracy rate 87.9% 86.4% of bipolar and 89.4% of schizophrenia correctly classified M(bp-hc): Average accuracy rate 59.8% 53.0% of bipolar and 66.7% of healthy controls correctly classified	Models based on gray matter density separated schizophrenia patients from healthy controls and bipolar disorder patients with high accuracy rate, and separated bipolar disorder from healthy control with much lower accuracy rate	
Marquand, Mourão-Miranda, Brammer, Cleare, & Fu, (2008)	Patients meeting criteria for major depression and in an acute episode of moderate severity with a minimum score of 18 on the 17-item HRSD Healthy controls with no history of psychiatric disorder, neurological disorder or head injury resulting in a loss of consciousness, and an HRSD score < 7 (*n* = 40)	Depression	To examine the sensitivity and specificity of the diagnosis of depression achieved with the neural correlates of verbal working memory	Machine learning algorithms	SVM	DSM-IV criteria for major depression HRSD	fMRI data	Accuracy of 68% with sensitivity of 65% and specificity of 70% with the blood oxygenation level-dependent convolution model at the mid-level of difficulty, which corresponded to a distributed network of cerebral regions involved in verbal working memory	Functional neuroanatomy of verbal working memory provides a statistically significant but clinically moderate contribution as a diagnostic biomarker for depression	

Abbreviations: AUDIT: Alcohol Use Disorder Identification Test; ALSPAC: Avon Longitudinal Study of Parents and Children; AMT: Autobiographical memory test; AUROC and AUC: Area under the receiver operating characteristic curve; BACS: Brief Assessment of Cognition in Schizophrenia; BAI: Beck Anxiety Inventory; BIS-11: Barratt Impulsiveness Scale-11; BP: Background, medical history, and pregnancy/delivery variables; CAARMS: Comprehensive Assessment of At-Risk Mental State; CANTAB: Cambridge Neuropsychological Test Automated Battery; CNN: Convolutional Neural Network; CPTB: Copenhagen Psychophysiology Test Battery; C-SSRS: Columbia Suicide Severity Rating Scale; DART: Danish Adult Reading Test; DRF: Distributed Random Forests; DSM-IV: Diagnostic and Statistical Manual of Mental Disorder-IV; DT: Decision tree; DTI: Diffusion tensor imaging; EEG: electroencephalogram; EHR: Electronic Health Record; EMA: Ecological momentary assessment; EPDS: Edinburgh Postnatal Depression Scale; ERTC: Extremely randomized trees classifier; ETI-SF: Early Trauma Inventory-Short Form; EU-GEI: European Network of National Schizophrenia Networks Studying Gene–Environment Interactions Multimodal diagnostic accuracy; EXGB: Ensemble of extreme gradient boosting; fMRI: Functional magnetic resonance imaging; GBRT: Gradient Boosting Regression Trees; GHQ: General Health Questionnaire; HADS: Hospital Anxiety and Depression Scale; HAMD and HRSD: Hamilton Rating Scale for Depression; ICD: International Classification of Diseases; Koko: An online peer-to-peer crowdsourcing platform that teaches users cognitive reappraisal strategies that they use to help other users manage negative emotions; LASSO: Least absolute shrinkage and selection operator; LSTM: Long Short-Term Memory; MDD: Major Depressive Disorder; M.I.N.I.: Mini-International Neuropsychiatric Interview; MRI: Magnetic resonance imaging; p: p-value; NEURAPRO: A clinical trial conducted between March 2010 and the end of September 2014, tested the potential preventive role of omega-3 fatty acids in clinical high-risk participants; NLP: Natural Language Processing; NPV: Negative predictive value; PHQ: Patient Health Questionnaire; PPD: Postpartum depression; PPV: Positive Predictive Value; QoL: Quality of life; QOLm: Mental quality of life; RBC: Rank-biserial correlation; RF: Random Forest; RMSE: Root mean square error; RS: Resilience-14; SOC: Sense of Coherence-29; VPSQ: Vulnerable Personality Scale Questionnaire; SE: Regression coefficients; SF-36: 36-Item Short Form Survey; SIQ: Suicidal Ideation Questionnaire; sMRI: Structural magnetic resonance imaging; SOFAS: Social and Occupational Functional Assessment Score; SQ for KNHANES-SF: Stress Questionnaire for Korean National Health and Nutrition Examination Survey-Short Form; SVC and SVM: Support Vector Machine; SVM-RFE: Support Vector Machine Recursive Feature Elimination; UQ: Ubiquitous Questionnaire; WAIS III: Wechsler Adult Intelligence Scale® – Third Edition; W: Wilcoxon signed-rank test (one-sided) statistics; XRT: Extreme randomized forest.

### Monitoring

In the monitoring domain, a total of 40 articles, encompassing the paper under the diagnosis and monitoring categories, were incorporated, involving a cumulative participant count of 168,077. Table 3 shows that most studies (22/40 studies) focused on monitoring depression, with major depressive disorder being the mental health condition most frequently monitored (15/40 studies). The remaining studies monitored multiple psychiatric disorders such as anxiety (Jacobson et al., 2022; Zainal & Newman, 2024), personality disorders (Jacobson et al., 2022), schizophrenia (Brandt et al., 2023; Dong et al., 2024; Jacobson et al., 2022), bipolar disorder (Busk et al., 2020; Lee et al., 2021), multiple specific phobias (Hilbert et al., 2024), substance use disorder (Carreiro et al., 2024), and the comorbidity of depression and anxiety (Webb et al., 2022) (Table 3). Furthermore, there was one study on psychosis (Amminger et al., 2015), one on pediatric obsessive-compulsive disorder (Lenhard et al., 2018), and four articles discussed suicide (Barrigon et al., 2023; Choo et al., 2024; Rozek et al., 2020; Solomonov et al., 2021). In terms of research objectives, most studies focused on predicting treatment effectiveness or response (25/40) (Table 3). The studies monitored or predicted the effectiveness of pharmacological interventions, such as long-chain omega-3 fatty acids (Amminger et al., 2015), citalopram (Chekroud et al., 2016), and duloxetine (Maciukiewicz et al., 2018), using AI. One article addressed both the prediction of treatment effectiveness and the prognosis of mental health disorders during treatment (Chekroud et al., 2016). Some studies focused on predicting the effectiveness of psychotherapy, such as cognitive behavioral therapy (CBT; Lenhard et al., 2018) and response to repetitive transcranial magnetic stimulation (Bailey et al., 2018; Dong et al., 2024). Some biomarkers, as well as sociodemographic (Chekroud et al., 2016; Kautzky et al., 2018; Vitinius et al., 2019), somatic (Vitinius et al., 2019), and emotional (Dougherty et al., 2023) data, predicted treatment outcomes or were used to generate predictive models of treatment-resistant depression (Kautzky et al., 2018). The effectiveness of prediction with online screening tools was also evaluated (Athreya et al., 2021). All articles in the monitoring domain used machine learning models, with the most commonly used models including random forest (Bao et al., 2021; Brandt et al., 2023; Hammelrath et al., 2024; Hilbert et al., 2024; Jacobson et al., 2022; Kautzky et al., 2018; Lenhard et al., 2018; Nie et al., 2018; Solomonov et al., 2021; Van Bronswijk et al., 2021; Wang, Wu, et al., 2024; Zainal & Newman, 2024; Zou et al., 2023), support vector machine (Bailey et al., 2018; Bao et al., 2021; Browning et al., 2019; Carreiro et al., 2024; Furukawa et al., 2020; Guilloux et al., 2015; Lenhard et al., 2018; Maciukiewicz et al., 2018; Wang, Wu, et al., 2024; Weintraub et al., 2023; Zainal & Newman, 2024; Zou et al., 2023), and elastic net regularization, a statistical technique that combines the penalties of Least Absolute Shrinkage and Selection Operator (LASSO) and ridge regression to effectively handle multicollinearity and perform variable selection in high-dimensional datasets (Wu et al., 2022).

**Table 3.** Studies on AI-assisted monitoring in mental health

Ref.	Subject description	Mental health condition	Aim	AI-based method	Models	Variables for monitoring/prediction	Results and accuracy	Conclusions
Carreiro et al. (2024)	Patients with substance use disorder (*n* = 30)	Stress Craving	To uses continuous physiologic data to detect high-risk behavioral states (stress and craving) during substance use disorder recovery	Machine learning models	DT Discriminant analysis LR Naive Bayes classifiers SVM Nearest neighbor classifiers Ensemble classifiers	Time-series raw physiologic data from the commercial sensor Basic demographics, phone and operating system information, current medications, and self-reported past medical, mental health, and substance use history	Stress detection AUC: 0.78 Craving detection AUC: 0.74 Stress vs Craving detection AUC: 0.75	All models performed close to previously validated models from a research grade sensor
Choo et al. (2024)	People with borderline personality disorder (*n* = 80)	Suicidal ideation	To explore predicting suicidal ideation in individuals with borderline personality disorder using EMA data	Machine learning models	MEM RNN	Baseline: Sex, any prior suicide attempt, baseline BDI, Affective Lability Scale, MDD diagnosis, BSSI, Barrett Impulsivity Scale, Childhood Trauma Questionnaire, and HDRS EMA: Suicidal ideation, stressful events, coping strategies, affect items, and suicidal behavior	MEM (RMSE = 3.84, MAPE = 56%, pseudo-R^2^ = 16%) RNN (RMSE = 3.41, MAPE = 42%, pseudo-R^2^ = 26%)	RNN showed enhanced predictive accuracy for higher SI values and participants with depression diagnoses or higher baseline depression score
Dong et al. (2024)	Patients with diagnosis of schizophrenia (*n* = 92)	Schizophrenia	To predict the responsiveness of patients with schizophrenia to rTMS treatment	Machine learning models	Base model Stacker model Sequential model	16 clinical variables (e.g., PANSS, CDSS, CGI, GAF, MADRS) 4 comorbidity variables (lifetime history of alcohol abuse, alcohol addiction, substance abuse, substance addiction prior to study recruitment) 5 sociodemographic variables (marital status, employment status, housing status, education, sum of education years from parents) PRS sMRI imaging data	Balanced accuracy for predicting ≥20% reduction in negative symptoms of PANSS: Active treatment group: 94% Sham treatment group: 50%	Key predictors of non-response: Clinical + PRS model: Apparent sadness, inability to feel, education level, unemployment sMRI model: Gray matter density reductions in default mode network, limbic networks, cerebellum Sequential modeling approach enhanced predictive accuracy while reducing diagnostic complexity
Hammelrath et al. (2024)	Patients with mild-to-moderate depression (training sample: *n* = 1270; test sample: *n* = 318)	Mild-to-moderate depression	To compare algorithms using features collected at baseline or early in treatment to predict non-response to a 6-week online depression program	Machine learning algorithm	RF	Baseline variables: Sociodemographic variables(e.g., age, sex, marital status, education, occupation, BMI) Processing (registration year, study variant, treatment affected by the corona pandemic) Healthcare system usage (e.g., previous treatment, usage during the last 4 weeks) Clinical variables (e.g., SCID, BDI-II, PHQ-Depression, COSTA, QoL) Variables of early treatment (week 2) PHQ-Depression COSTA SEWIP	Best performance form early treatment variables AUC: 0.71–0.77 Recall: 0.75–0.76	Therapeutic alliance and early symptom change constituted the most important predictors
Hilbert et al. (2024)	Patients with a diagnosis of panic disorder, agoraphobia, social anxiety disorder, or multiple specific phobias (*n* = 309)	Panic disorder, agoraphobia, social anxiety disorder, or multiple specific phobias	To test if functional neuroimaging data maintains strong prediction accuracy in larger samples using rs-fMRI data	Machine learning models	RF LR Majority voting Softmax voting Weighted softmax voting	Clinical and demographic variables (e.g., age, sex, baseline severity) Resting state-fMRI data (ROI-to-ROI and edge-functional connectivity, sliding-windows, and graph measures)	Accuracy: 0.465–0.600 Balanced accuracy: 0.465–0.613 Sensitivity: 0.460–0.687 Specificity: 0.375–0.539	Caution is advised when interpreting promising prediction results from neuroimaging data in small samples
Wang, Wu, et al. (2024)	College students with symptoms of anxiety or depression (*n* = 107)	Symptoms of anxiety, depression, stress	To predict efficacy and response using machine learning in college students undergoing biofeedback therapy	Machine learning model	ANN	Heart rate variability characters (time and frequency domains) Acoustic variables from the data using a speech frame (32 ms)	Model accuracy for anxiety treatment response: 62%	Speech features, such as the energy parameters as more accurate and objective indicators for tracking biofeedback therapy response and predicting efficacy
Wang, Wu, et al. (2024)	Patients with MDD (training samples: *n* = 85; test samples: *n* = 147)	MDD	To predict treatment response by using neuroimaging data	Machine learning models	RF GBDT XGBoost Penalized LR SVM Neural network	307 brain imaging variables 49 questionnaire variables from QIDS and HDRS (including baseline and week 8 HDRS scores) 4 clinical and demographic variables (age, total years of education, sex, and medication use)	Training set model AUC: 0.615–0.8257 Testing set model AUC: 0.4884–0.4941	The machine learning pipeline exhibited high accuracy and AUC (>0.80) on the training set but encountered challenges when applied to an external validation dataset, prompting an investigation into site heterogeneity issues
Zainal and Newman (2024)	Patients with GAD (*N* = 110)	GAD	To identify which clients with generalized anxiety disorder benefit from mindfulness ecological momentary intervention versus self-monitoring app	Machine learning models	LR SVM - radial kernel RF	Demographic variables (i.e., age, gender, and race/ethnicity) GAD-Questionnaire-IV FFMQ Wechsler Adult Intelligence Scale–Fourth Edition Controlled Oral Word Association Test	GAD severity prediction SVM nested leave-one-out cross-validation: AUC = 0.817, accuracy = 0.800, balanced accuracy = 0.795, sensitivity = 0.767, specificity = 0.822 RF nested leave-one-out cross-validation: AUC = 0.817, accuracy = 0.819, balanced accuracy = 0.814, sensitivity = 0.791, specificity = 0.837	Predictors of optimization to the intervention were higher anxiety severity, higher trait perseverative cognition, lower set-shifting deficits, older age, and stronger trait mindfulness
Brandt et al. (2023)	Participants with schizophrenia or schizoaffective disorder (aged ≥18 years) (*n* = 1392)	Schizophrenia or schizoaffective disorder	To identify general prognostic factors of relapse for all participants (irrespective of treatment continuation or discontinuation) and specific predictors of relapse for treatment discontinuation	Machine learning	Proportional hazard regression model (for multivariate analysis) Random survival forests (for exploratory analysis to improve the predictive ability)	36 variables: Demography (sex, age) Somatic history (somatic illness, BMI) Psychiatric history (e.g., disorganized type, catatonic type, paranoid type, residual type, duration of illness) Substance use (smoking, drug-positive urine screening) Standardized scales (PANSS, CGI, AIMS, BARS, PSP) Treatment characteristics before randomization (e.g., last dosage of the antipsychotic study drug, treatment duration of antipsychotic study drug) Comedication Adverse events Laboratory results (e.g., alanine aminotransferase, prolactin, white blood cell count)	The concordance index for predictive performance was 0.707, meaning that the algorithm’s prediction about which of the two participants will relapse sooner is correct in 71% of the cases	Out of the 36 baseline variables, general prognostic factors of increased risk of relapse for all participants were drug-positive urine; paranoid, disorganized, and undifferentiated types of schizophrenia; psychiatric and neurological adverse events; higher severity of akathisia; antipsychotic discontinuation; lower social performance; younger age; lower glomerular filtration rate; benzodiazepine comedication Predictors of increased risk specifically after antipsychotic discontinuation were increased prolactin concentration, higher number of hospitalizations, and smoking
Barrigon et al. (2023)	Patients with a history of suicidal thoughts and behavior (*n* = 225)	Suicidal ideation	To predict short-term (one week) suicide risk by using smartphone data in suicidal patients	Machine learning algorithm	Bayesian algorithm	Distance traveled Time spent at home Steps taken Use of any app	AUC: 0.78	Unsupervised machine learning on smartphone data from patients with suicidal ideation effectively predicts suicide risk
Dougherty et al. (2023)	Patients with TRD (*n* = 233)	TRD	To predict which participants with treatment-resistant depression would be week 3 responders and sustained responders through week 12 to psilocybin treatment	Machine learning algorithms and models	NLP LR	Two-dimensional sentiment from the first session (computed by NLP), emotional breakthrough index, treatment dose	At week 3: Accuracy: 85% AUC: 88% At week 12: Accuracy: 88% AUC: 85%	Treatment response to psilocybin is accurately predicted using a logistic regression model incorporating NLP metrics, EBI scale responses, and treatment arm data
Harrer et al. (2023)	Patients with chronic back pain and depressive symptoms (*n* = 504)	Depressive symptoms	To predict treatment effects of an Internet-based depression intervention for patients with chronic back pain	Machine learning models	DT (developed by multilevel model-based recursive partitioning)	Sociodemographic variables (e.g., age, gender, marital status, education, internet affinity, social support, medication, sick leave) Symptom severity and quality of life (PHQ–9, HAMD, NPRS, QoL) Pain-related risk factors (PSEQ, ODI, SPE)	Decision tree model: R^2^app = 52% After bootstrap bias-correction, R^2^adj = 45% During external validation, R^2^adj = 33%	Predictions of the multivariate tree learning model suggest a pattern in which patients with moderate depression and relatively low pain self-efficacy benefit most, while no benefits arise when patients’ self-efficacy is already high
Jankowsky et al. (2024)	Naturalistic inpatients (*n* = 723)	Anxious and depressive symptoms	To compare machine learning algorithms for predicting treatment response in naturalistic inpatient samples	Machine learning algorithms	Linear regression EN regression Gradient boosting machines	Sociodemographic background variables (e.g., gender, age) Indicators of physical health (e.g., subjective health, BMI, smoking) Indicators of personality and mental health (e.g., maladaptive personality traits, anxiety or depression scores) Treatment variables (e.g., number of treatments within the last 12 months)	Training: R^2^: 0.329–0.70 Test: R^2^: 0.315–0.441	Treatment-related variables were the most predictive, followed psychological indicators
Ricka et al. (2023)	Patients with MDD (*n* = 26)	MDD	To identify markers of mood disorders using six months of physiological and clinical data by machine learning	Machine learning algorithm	Label extension and detrending processes, a feature selection, and a deep learning multilayer perceptron model	Physical activity (12 variables) Heart rate (25 variables) Heart rate variability (39 variables) Breathing rate (12 variables) Sleep (13 variables) MADRS scores	2-class prediction (depressed/not depressed) Accuracy: 86% Sensitivity: 79% Specificity: 94%	A supervised ML system can efficiently predict a patient’s clinical score by identifying their biosignature of symptoms during a MDD episode
Scodari et al. (2023)	Patients with subclinical depression (*n* = 236)	Minor depressive symptoms	To forecast symptom changes among subclinical depression patients receiving stepped care or usual care	Machine learning models	Tree-based and nested framework	15 categorical variables: Gender, marital status, parental birthplace, rural residential area, employment status, education level, excessive alcohol usage, current smoking behavior, normal exercise behavior, onset of depression, baseline dysthymia status, and presence of comorbid illness, and comorbid conditions 8 continuous variables: The number of chronic diseases, BMI, number of historical depressive episodes, baseline locus of control, baseline social support, baseline HADS scores, baseline PHQ–9 scores	For the intervention group, the R^2^ for models at various treatment time intervals are as follows: 0–3 months: 0.15 0–6 months: 0.13 0–9 months: 0.21 0–12 months: 0.12 For the usual care group, the R^2^ for models at different treatment time intervals are as follows: 0–3 months: 0.24 0–6 months: 0.15 0–9 months: 0.15 0–12 months: 0.11	Patients who received stepped care were more likely to reduce PHQ–9 scores if they had high PHQ–9 but low HADS-Anxiety scores at baseline, a low number of chronic illnesses, and an internal locus of control
Zou et al. (2023)	Patients with MDD (*N* = 245)	MDD	Using passive sensing data to predict treatment response in patients with MDD	Machine learning models	SVM LR RF LSTM GRU GRU-Decay	Call log (type of phone call, mean and SD time of all calls being made, mean and SD of duration, number and entropy of phone calls) Phone usage (frequency and duration of smartphone usage in a day, duration of phone usage for each period (6–12 pm, 12–6 pm, 6–0 am), earliest and latest phone usage time) App usage (duration of social apps, content-providing apps, shopping apps, and entertainment apps) Sleep and step data (duration and ratio of both light and deep sleep, wake-up and sleep times)	GRU-Decay Precision: 0.61 Recall: 0.64 F1 score: 0.58 AUC: 0.65 Other models Precision: 0.57–0.71 Recall: 0.22–0.59 F1 score: 0.33–0.54 AUC: 0.54–0.59	In terms of recall, F1 score, and AUC, the sequence model based on GRU-Decay achieve the best performance
Weintraub et al. (2023)	Youth aged 13to 19 who had active mood symptoms, mood instability, and at least one parent with bipolar or MDD (*n* = 44)	Depressive symptoms	Use of machine learning to identify the speech features that most strongly correlated with concurrent depressive symptoms over 18 weeks	Machine learning algorithm	SVM	PSRs from the Adolescent Longitudinal Interval Follow-up Evaluation 20 speech features reflecting affective processes, social processes, drives, informal, time orientation words etc.	Strongest correlated combination of features: affective processes, drives, informal, leisure, and risk (r = 0.47, 95% CI: 0.37–0.56, R^2^ = 0.12) Strongest association of features from subject’s first speech features: affective processes, nonfluencies, drives, and risks (r = 0.68, 95% CI: 0.48–0.81, R^2^ = 0.11)	Speech features identified by machine learning analysis achieved moderate correlation
Jacobson et al. (2022) Note: Also included in the diagnosis domain	Participants aged 38.5 years old on average (*n* = 126,060)	MDD Generalized anxiety disorder Social anxiety disorder Panic disorder Borderline personality Paranoid personality disorder Schizophrenia	To examine the effectiveness of prediction of mental health outcomes based on exposure to online screening tools	Machine learning	RF Cox Proportional Hazards Models	Screening tool topic Screening tool attributes Hour of the day and day of the week at which the screening tool was clicked Whether the screening tool was a Mental Health America screening tool or from another online web domain Number of previous searches which resulted in a click to a screening tool Past interests, e.g., distribution of query topics prior to the clicking on the first screening tool by each use (to ascertain whether the online screen information provided incremental information to their general search pattern types)	Prediction accuracy was high for mental health self- references, self-diagnosis, and seeking care: screen content predicted later searches with mental health self-references (AUC =0·73), mental health self-diagnosis (AUC = 0·69), mental health care-seeking (AUC = 0·61) Other outcomes were more difficult to predict: psychoactive medications (AUC = 0·55), suicidal ideation (AUC = 0·58), and suicidal intent (AUC = 0·60) Cox proportional hazards models suggested individuals utilizing tools with in-person care referral were significantly more likely to subsequently search for methods to actively end their life (HR = 1·727)	Online screens may influence help-seeking behavior, suicidal ideation, and suicidal intent Websites with referrals to in-person treatments could put persons at greater risk of active suicidal intent
Nguyen et al. (2022)	Participants with MDD, included early onset (before the age of 30) and chronic (episode duration of two years) or recurrent (2+ episodes) disease episodes (*n* = 222)	MDD	To determine whether pretreatment reward task-based fMRI can predict treatment-specific outcome	Deep learning models	Feedforward neural network (a separate model was trained for each treatment: sertraline, bupropion, and placebo)	Reward task-based fMRI, which was acquired during a block-design number-guessing task that probes reward processing neural circuitry known to be altered in MDD Clinical measurements Demographic features	For predicting change in HAMD Model 1 on sertraline R^2^: 48%; RMSE: 5.15 Model 2 on bupropion R^2^: 34%; RMSE: 4.46 Model 3 on placebo R^2^: 28%; RMSE: 5.87	All the models explained a substantial proportion of the variance in change in HAMD. The combination of these predictive models presented a possible precision medicine approach for antidepressant selection, and each model would be applied to provide a prediction of response to each treatment
Webb et al. (2022)	School district employees aged 18 or above who owned a smartphone, had limited exposure to meditation app, and had depressive symptoms below the severe range (*n* = 662)	Depression and anxiety	To use a data-driven algorithm to predict which individuals are most likely to benefit from app-based meditation training	Machine learning	ENRR	Pre-intervention distress, anxiety, depression, stress, repetitive negative thinking, the mindfulness aspect of acting with awareness, loneliness, diffusion, presence, search for meaning, self-compassion, well-being, age, gender, race, marital status, and income Anxiety measure, PROMIS Depression measures, and 10-item Perceived Stress Scale	Multivariable ENRR model: Higher baseline levels of the following variables predicted a greater reduction in distress: distress (r = −0.30) depression (r = −0.30) stress (r = −0.26) Higher baseline scores of the following variables predicted greater reduction in distress in the control condition: diffusion, presence, distress, anxiety, stress, depression, and loneliness Linear regression mode: Higher levels of repetitive negative thinking predicted: a greater reduction in distress from the mindfulness app (B = −0.02) higher levels of repetitive negative thinking were significantly associated with poorer outcomes in the control condition (B = 0.01) Overall: A significant group with PAI interaction was observed linear regression model including repetitive negative thinking as the sole baseline predictor: r2 = 0.11 multivariable ENRR model: r2 = 0.10	Either the linear regression model with a single predictor of baseline levels of repetitive negative thinking, or the multivariable ENRR model with multiple predictors can predict changes in the level of distress
Athreya et al. (2021)	People with nonpsychotic MDD and received at least 8 weeks of treatment with a study drug, including SSRIs, SNRIs or TCAs, placebo (*n* = 3,518)	Depression	To identify specific depressive symptoms and thresholds of improvement that were predictive of antidepressant response	Machine learning	Gaussian mixture models Probabilistic graphical models	Four HDRS items (depressed mood, psychic anxiety, guilt feelings/ delusions, and work/activities) Thresholds of change in prognostic symptom severity, derived based on the absolute difference in median scores on symptom dynamic paths between baseline and four-week strata	Four depressive symptoms and specific thresholds of four-week change in each symptom predicted the eventual eight-week outcome of SSRI therapy with an average accuracy of 77%. The symptoms and thresholds derived from patients treated with SSRIs correctly predicted outcomes in 72% of patients treated with other antidepressants	Conjunction of the two AI models derived consistently high predictive accuracies across numerous commonly prescribed antidepressants, and hence interpretable and accurate prognoses of antidepressant treatment outcomes
Bao et al. (2021)	Depressive patients receiving six intravenous infusions of ketamine over 2 weeks (*n* = 83)	MDD	To identify a set of biomarkers that could be used to predict clinical outcomes for treatment in MDD	Machine learning	SVM RF kNN LR DT LR with EN	Age, sex, BMI, smoking status, and the HAMD score	Accuracy: SVM: 0.62 ± 0.23 RF: 0.56 ± 0.15 kNN: 0.63 ± 0.12 LR: 0.62 ± 0.12 DT: 0.57 ± 0.12 LR with EN: 0.63 ± 0.19	Machine learning approach could predict treatment outcomes of multiple ketamine infusions on the basis of the genotyping information
Lee et al. (2021)	Adults aged 18 to 65 with bipolar disorder (*n* = 60)	Bipolar depression	To identify biologically relevant moderators of response to TNF-α inhibitor, infliximab	Machine learning	CART	Plasma cytokine and neuronal origin-enriched extracellular vesicle protein concentrations, intervention assignment and week SHAPS MADRS	Accuracy of predicting reduction in anhedonic symptoms with baseline cytokine biotype, intervention allocation, week, and baseline and change in neuronal origin-enriched extracellular vesicle factor scores: r^2^ = 0.22 RMSE = 0.08 No significant moderation effect is observed in MADRS total score by baseline biotype	Pretreatment biotypes, which derived from peripheral cytokine measurements, can predict antianhedonic efficacy with infliximab
Solomonov et al. (2021)	Older adults over 60 who suffered from unipolar, nonpsychotic MDD (*n* = 221)	Suicidal ideation	To identify baseline predictors of the course of suicidal ideation	Machine learning algorithms	LASSO RF GBM Classification tree	Demographics, treatment assignment, age of onset, length of current episode, number of previous episodes, severity of depression, disability, cognitive impairment, executive functioning, neuroticism, apathy, hopelessness, activation, avoidance/rumination, work/school impairment avoidance/rumination; social impairment; anhedonia; rumination response style scale; and digit span	Predictive performance: LASSO: AUC = 0.735 GBM: AUC = 0.725 RF: AUC = 0.684 Classification tree: AUC = 0.670	Four machine learning algorithms identified hopelessness, neuroticism, and low general self-efficacy as the strongest predictors of an unfavorable trajectory of suicidal ideation
Van Bronswijk et al. (2021)	Adult outpatients recruited from the mood disorders unit with a primary diagnosis of MDD (*n* = 151)	MDD	To extend the PAI to long-term depression outcomes after acute-phase psychotherapy	Two-step machine learning	RF Regression model	38 pretreatment variables from six domains: depression variables demographics psychological distress general functioning psychological processes life and family history	For parental alcohol abuse, the regression coefficients across the bootstrapped samples were stable with a positive value in 99.8% of the samples	A history of parental alcohol abuse was associated with higher BDI-II scores during the 17-month follow-up phase. Therefore, parental alcohol abuse could be used as a predictor for long-term depression outcomes following cognitive therapy and interpersonal psychotherapy
Busk et al. (2020)	Patients with bipolar disorder who had previously been treated (*n* = 15,975)	Bipolar disorder	To examine the feasibility of forecasting daily subjective mood scores based on daily self-assessments	Multi-task learning	Hierarchical Bayesian models	Daily self-assessments via Android smartphone app, including activity, alcohol, anxiety, irritability, cognitive difficulty, medicine intake, presence of mixed mood, mood, sleep, stress Clinical evaluations with HDRS and YMRS to assess depression and mania	Historical mood was the most important predictor of future mood, with self-reported mood scores and HDRS scores were negatively correlated (r = −0.40) whereas self-reported mood scores and YMRS scores were positively correlated (r = 0.22)	Application of hierarchical Bayesian models could forecast subjective mood for up to 7 days, thus improving continuous disease monitoring
Furukawa et al. (2020)	Patients aged 25 to 75 years, with nonpsychotic unipolar MDD episode, and having received no antidepressant, antipsychotic, or mood stabilizer in the previous month (*n* = 2,011)	MDD	To predict depression severity from a large set of baseline predictors through a web app	Machine learning	Penalized linear regression models using LASSO Penalized linear regression models using the ridge penalty SVM with a polynomial or radial kernel Artificial neural networks with one hidden layer, three or four nodes	Sociodemographic variables including age, sex, education, employment status, and marital status Baseline clinical characteristics include age at onset of depression, number of previous depressive episodes, length of index episode, and concurrent physical illness Depression characteristics by week three include individual item scores of PHQ–9 for the index episode; individual item scores of the BDI-II; individual item scores of the FIBSER; and adherence to pharmacotherapy	SVMs are observed with a lower prediction error in both internal and internal-external cross-validation (MAE = 1.5)	Three different SVMs with a radial kernel, one SVM per treatment arm, could be chosen to predict treatment outcome
Rajpurkar et al. (2020)	Outpatients aged 18 to 65 from primary or specialty care practices with a diagnosis of MDD (*n* = 518)	MDD	To identify the extent to which a machine learning approach can predict acute improvement for individual depressive symptoms with antidepressants based on pretreatment symptom scores and EEG measures	Machine learning	ELECTREE Score algorithm using GBDTs	Resting-state EEG continuously recorded Symptoms of HRSD–21	C index score, which is indicative of discriminative performance, was found for 12 symptoms. The highest C index score was found on: loss of insight (C index, 0.963 [95% CI 0.939–1.000]) unreality and nihilism (C index, 0.951 [95% CI, 0.932–0.976]) weight loss (C index, 0.923 [95% CI, 0.896–0.953]) The most critical predictor for each symptom was the baseline symptoms severity Any single EEG feature was higher than 5% predictors for seven symptoms Combination of EEG and baselines symptom feature significantly increased the C index for improvement in four symptoms: Energy loss (C index increase, 0.035 [95% CI, 0.011–0.059]) Appetite changes (C index increase, 0.017 [95% CI, 0.003–0.030]) Psychomotor retardation (C index increase, 0.020 [95% CI, 0.008–0.032]) Loss of insight (C index increase, 0.012 [95% CI, 0.001–0.020])	The machine learning model could predict the improvement in depressive symptoms most accurately with baseline symptom severity in combination with EEG features
Rozek et al. (2020)	Army soldiers reporting active suicide ideation with intent to die during the previous week and/or a suicide attempt during the previous month (*n* = 152)	Suicide	To examine predictors of suicidal behaviors among high-risk suicidal soldiers who received outpatient mental health services in a RCT of Brief CBT for Suicide Prevention compared to treatment as usual	Machine learning	MondoBrain Augmented Intelligence® System	BSSI-W Prior attempts Treatment Group SCS Sex	This combination of variables correctly classified eight of 26 participants who attempted suicide during the two-year follow-up period (30.8%) and misclassified only one of 126 participants who did not attempt suicide (0.8%), yielding 88.9% positive predictive value, and 87.4% negative predictive value	This combination of variables correctly classified almost one-third of participants who attempted suicide in the subsequent two years with good positive predictive value and negative predictive value
Browning et al. (2019)	Depressive patients whose treating clinician had made the decision to prescribe citalopram (*n* = 239)	Depression	To assess whether changes in emotional processing and subjective symptoms over the first week of antidepressant treatment predicts clinical response after four–eight weeks of treatment	Machine learning	SVM	QIDS-SR16, ECAT, EREC, FERT	Accuracy: QIDS-SR16: ~60% FERT: 70% ECAT & EREC: 50–60% QIDS-SR16 & FERT: 77% QIDS-SR16, FERT, ECAT & EREC: 79%	Cognitive and symptomatic measures were possible to be used in guiding antidepressant treatment in depressed patients
Foster et al. (2019)	Adolescents aged 12–17 with MDD (*n* = 439)	MDD	To estimate patient-specific inter-treatment differences among three treatment conditions: CBT, FLX, and the combination of CBT and FLX, as a function of patients’ baseline characteristics	Machine learning	Model-based Random Forest	Gender, race, family income, referral source, dysthymia, anxiety disorder, ADHD, childhood trauma, study site, age, verbal intelligence, current episode duration, baseline depression severity, functional impairment, suicidal ideation, melancholic features, number comorbid diagnoses, caregiver depression, conflict with caregiver, hopelessness, cognitive distortions, treatment expectations from parent, treatment expectations from adolescents	FLX-CBT difference: FLX was more effective (b = −0.13, 95% CI: −0.22 to −0.05), especially with more severe baseline depression CB -combination difference: Combination was more effective (b = −0.25, 95% CI: −0.33 to −0.17) FLX-combination difference: Combination was more effective (b = −0.11, 95% CI: −0.21 to −0.02), especially with less severe baseline depression and higher treatment expectations from patients	Combined treatment with CBT and FLX was consistently superior to either therapy administered alone across a broad range of patients
Vitinius et al. (2019)	Depressed patients with CAD (*n* = 570)	Depression	To identify somatic and sociodemographic predictors of depression outcome among depressed patients with CAD	Machine learning	LR and linear or binomial linear model with LASSO regularization	141 potential sociodemographic and somatic predictors including blood tests, medical history, current drug use, comorbidities, and sociodemographic data. HADS	Predictors to favorable depression outcome: higher heart rate variability during numeracy tests (p = 0.020), unknown previous myocardial infarction (p = 0.013), higher age (p = 0.002) Predictors to unfavorable depression outcome: anticholinergic drugs (p = 0.045), state after resuscitation (p ≤ 0.042), uric acid drugs (p ≤ 0.039), beta blockers (p = 0.035), New York Heart Association (NYHA) class III (p ≤ 0.028), analgesic drugs (p = 0.027), antidiabetic drugs (p = 0.015), higher triglycerides (p = 0.014), intake of thyroid hormones (p = 0.007), and hyperuricemia (p ≤ 0.003)	Machine learning could identify somatic and sociodemographic predictors of depression outcome in patients with CAD
Bailey et al. (2018)	Patients with TRD and healthy controls aged 20 to 72 with normal or corrected to normal vision (*n* = 50)	Depression	To determine whether working memory related power, connectivity, and theta- gamma coupling measures could be used to predict responders to rTMS treatment for treatment-resistant depression	Multivariate machine learning	SVM	Mood: Montgomery-Asberg depression rating scale Behavior: working memory accuracy, average reaction time EEG: alpha, theta, and gamma power, connectivity, and theta-gamma coupling	Prediction of individual responders: mean sensitivity: 0.91 (±0.06 SD) specificity: 0.92 (±0.02 SD) balanced accuracy: 91% (±3.64 SD)	Baseline and week 1 frontal-midline theta power and theta connectivity showed good potential for predicting response to rTMS treatment for depression
Kautzky et al. (2018)	Patients diagnosed with MDD (*n* = 55)	MDD	To generate a prediction model for TRD using machine learning featuring a large set of clinical and sociodemographic predictors of treatment outcome	Machine learning	RF	47 predictors documented in the GSRD database, which can be classified into: Sociodemographic MDD history Axis II comorbidity Axis III comorbidity Clinical features Other predictors, e.g., inpatient or outpatient, quality of social life, quality of work life, quality of family life, retrospective MADRS score	The full model with 47 predictors yielded an accuracy of 75.0% for predicting TRD and treatment response, with positive predictive value of 79.6%, and negative predictive value of 67.9% When the number of predictors was reduced to 15, accuracies between 67.6% and 71.0% were attained for different test sets	Machine learning techniques have shown promising results on prediction of TRD by considering interaction and main effects equally and producing reliable classification with high accuracy
Lenhard et al. (2018)	Adolescents with aged 12–17 with OCD and had received either immediate or delayed (12 weeks) internet-delivered CBT (*n* = 61)	Pediatric OCD	To test four different machine learning methods in the prediction of treatment response in a sample of pediatric OCD patients who had received internet-delivered CBT	Machine learning	Linear model with best subset predictor selection L1 Elastic Net (LASSO) RF SVM	46 demographic and clinical baseline variables, related to: Parental education level Referral to study Medication Previous treatment experience Comorbidity Number of comorbid diagnoses Baseline OCD symptoms Clinical Global Impression Self-rated baseline measures Parent-rated baseline measures Outcome at posttreatment Outcomes at three-month follow-up	Accuracy: Linear model with best subset predictor selection: 83% L1 Elastic Net (LASSO): 75% RF: 75% SVM: 75%	Machine learning models were able to predict treatment outcome in internet-delivered CBT for pediatric OCD with good to excellent accuracy
Maciukiewicz et al. (2018)	Individuals diagnosed with MDD from three clinical trials who received duloxetine or placebo for up to eight weeks (*n* = 186)	MDD	To use supervised machine learning to build predictive models of duloxetine outcome for MDD with genome-wide data	Machine learning models	LASSO regression CRT SVM	SNPs	Accuracy on remission prediction: CRT = 0.51 SVM = 0.52 Accuracy on prediction of treatment response accuracy: CRT = 0.57 SVM = 0.64 (chance accuracy = 0.57) Of the 19 most robust SNPs, 17 were characterized by large LASSO coefficients	None of the machine learning models performed satisfactorily in remission prediction. For treatment response, SVM achieved moderate performance whereas CRT’s performance was just equal to chance accuracy
Nie et al. (2018)	STAR*D cohort: Patients with MDD. RIS-INT–93 cohort: Patients with MDD and had history of resistance to therapy with antidepressant medication and were treated prospectively with citalopram for up to six weeks (*n* = 5686)	MDD	To identify risk factors of treatment resistance by extending the work in predictive modeling of treatment-resistant depression via partition of the data from the STAR*D cohort and completely independent cohort RIS-INT–93 into training and testing datasets	Machine learning	l_2_ penalized LR RF GBDT XGBoost EN	CRS, demographics, PHX, MHX, PRISE, PDSQ, baseline and week two of level 1 treatment which include records from Clinic Visit Form, QIDS-C_16_, QIDS-SR_16_, Bech melancholia scale, the Maier-Phillipp severity subscale, the Santen Subscale, the Gibbons’ global depression severity scale, HAM-D_7_	STAR*D testing dataset and RIS-INT–93 independent dataset with an AUC of 0.70–0.78 and 0.72–0.77, respectively	The series of machine learning models were able to predict treatment-resistant depression using clinical and sociodemographic data
Chekroud et al. (2016)	STAR*D trial: Patients from primary and psychiatric care settings, with nonpsychotic MDD, with at least 14 score on 17-item HAMD, and aged 18–75 COMED trial: Patients with nonpsychotic MDD, had recurrent or chronic depression, with at least 16 scores on 17-item HAMD, and aged 18–75 (*n* = 4041)	MDD	To develop an algorithm to assess whether patients will achieve symptomatic remission from a 12-week course of citalopram	Machine learning	EN	Overlapping variables in the two clinical trials including sociodemographic features, DSM-IV-based diagnostic items, depressive severity checklists, eating disorder diagnoses, whether the patient had previously taken specific antidepressant drugs, the number and age of onset of previous major depressive episodes, and the first 100 items of the psychiatric diagnostic symptom questionnaire	Accuracy in internal validation: STAR*D cohort: 64.6% Accuracy in external validation: COMED cohort (escitalopram treatment group): 59.6% COMED cohort (escitalopram-bupropion treatment group): 59.7% COMED cohort (venlafaxine-mirtazapine treatment group): 51.4%	Machine learning achieved moderate performance for internal prediction. The performance across cohort varied for different treatment groups showed fair to moderate accuracy
Iniesta et al. (2016)	Treatment-seeking adults with MDD and a current depressive episode (*n* = 793)	MDD	To optimize prediction of symptom improvement and remission during treatment with escitalopram or nortriptyline	Machine and statistical learning	ENRR	Demographics data including current age, age at onset of depression, sex, smoking status, BMI, occupation, marital status, years of education and number of children Baseline severity measures including the clinician-rated MADRS, the 17-item HRSD and the self-report BDI Individual depressive symptoms from the SCAN interview and depression subtypes Observed mood, cognitive and neurovegetative symptom factors, and six dimensions (mood, anxiety, pessimism, interest-activity, sleep, and appetite) from a published factor analysis Stressful life events experienced during the six months prior to the baseline assessment, measured with the LTE-Q Medication history included the use of antidepressant at the time of recruitment, any prior antidepressant treatment, number and types of antidepressants tried established with Medication History Form	Accuracy of prediction on different outcomes: Reduction in depressive symptoms: a model including 29 of the 60 predictors explained a 3.85% of the variance in MADRS scores change across treatment arms Remission: AUC = 0.72, R^2^ = 0.15 Predictors with strong contribution: Symptoms of depressed mood, reduced interest, decreased activity, indecisiveness, pessimism, and anxiety significantly predicted symptom improvement BMI, appetite, interest-activity symptom dimension, and anxious-somatizing depression subtype predicted remission	Easily obtained demographic and clinical variables could predict therapeutic response to escitalopram with clinically meaningful accuracy
Amminger et al. (2015)	Individuals with ultra-high risk for psychosis and meeting at least one operationally defined groups of risk factors for psychosis: Attenuated positive psychotic symptoms Transient psychosis Genetic risk plus a significant decrease in functioning (*n* = 81)	Psychosis	To determine biological and clinical factors associated with treatment response indexed by functional improvement in a pre–post examination of a 12-week intervention in individuals at ultra-high risk for psychosis	Machine learning	Linear regression models Gaussian Process Classification	Erythrocyte fatty acid composition of the phosphatidylethanolamine phospholipid fraction	Univariate analysis: Variance in prediction of functional improvement: In ω–3 PUFA group: ALA and negative symptoms explained 14% and 10% of the variance In-placebo group: Positive symptoms and functioning explained 23% and 11% of the variance Multivariate analysis: Overall accuracy of fatty acid prediction in treatment response: In ω–3 PUFA group: 86.7% In-placebo group: 79.2%	Univariate analysis: Higher levels of erythrocyte membrane ALA (parent fatty acid of the ω–3 family) and more severe negative symptoms at baseline predicted subsequent functional improvement in the treatment group Less severe positive symptoms and lower functioning at baseline were predictive on functional improvement in the placebo group Multivariate analysis: Fatty acids predicted response to treatment in both ω–3 PUFA and placebo groups with a high level of accuracy
Guilloux et al. (2015)	Anxious-depressed adults with nonpsychotic MDD episode of sufficient severity (score ≥ 15 on the 25-item HRSD) and elevated symptoms of panic or anxiety (score ≥ 7 on the past-month panic and agoraphobic spectrum self-report) Nonpatient controls not meeting criteria for any mood or anxiety disorder (*n* = 67)	MDD	To identify the biomarkers predicting nonremission prior treatment initiation	Machine learning prediction model	Random intercept model SVM	Peripheral blood-based gene expression	The results from these studies indicate an average cross-validated accuracy (i.e., model selection bias corrected) of 79.4% in predicting remission status, with the 13-gene model displaying the highest individual noncorrected prediction value (88%). The newly built prediction model in the validation cohort using the same 13 genes identified in the initial cohort, and found through another round of leave-one-out cross-validation that a 6-gene model achieved the highest accuracy (76.2%)	At pretreatment assessment, the gene expression profiles obtained from blood samples of MDD subjects who will not attain remission after treatment differ from nondepressed controls and also from MDD patients who will remit with treatment Six out of 13 genes identified in the initial cohort could predict remission in an independent cohort, which demonstrated the potential of pretreatment peripheral gene expression profiles to predict nonremission following an eight- to 12-week course of citalopram treatment

Abbreviations: ADHD: Attention-Deficit/Hyperactivity Disorder; AIMS: Abnormal Involuntary Movement Scale; ALA: α-linolenic acid; ANN: Artificial neural network; AUC: Area under the receiver operating characteristic curve; BARS: Barnes Akathisia Rating Scale; BDI: Beck Depression Inventory; BMI: Body mass index; BSSI-W: Beck Scale for Suicide Ideation, Worst Point; CAD: Coronary artery disease; CART: Classification and regression trees; CBT: Cognitive behavioral therapy; CDSS: Sum of Calgary Depression Scale for Schizophrenia; CGI: Clinical Global Impression; COSTA: Cognitive Style Assessment measuring cognitive distortions; CRS: Cumulative Illness Rating Scale; CRT: Classification and regression tree; DT: Decision tree; EBI: Emotional Breakthrough Index; ECAT: Emotional categorization task; EEG: Electroencephalographic; EMA: Ecological Momentary Assessment; EN: Elastic net; ENRR: Elastic net regularized regression; EREC: Emotional recall task; FERT: Face-based emotional recognition task; FFMQ: Five Factor Mindfulness Questionnaire; FLX: Fluoxetine; fMRI: Functional magnetic resonance imaging; GAD: Generalized anxiety disorder; GAF: Global Assessment of Functioning; GBDT: Gradient-boosted decision trees; GRU: Gated Recurrent Unit; GSRD: Group for the Study of Resistant Depression; HADS: Hospital Anxiety and Depression Scale; HAMD: Hamilton Depression Rating Scale; HDRS: Hamilton Depression Rating Scale; HRSD: Hamilton Rating Scale for Depression; kNN: K-nearest neighbor; LASSO: Least absolute shrinkage and selection operator; LR: Logistics regression; LSTM: Long Short-Term Memory; LTE-Q: List of Threatening Experiences Questionnaire; MADRS: Montgomerye-Åsberg Depression Rating Scale; MAPE: Mean absolute percent error; MDD: Major depressive disorder; MEM: Mixed-effects linear regression models; MHX: Medication history; NLP: Natural language processing; NPRS: Numerical pain rating scale; ODI: Oswestry Disability Index; OCD: Obsessive-compulsive disorder; PAI: Personalized Advantage Index; PANSS: Positive and Negative Syndrome Scale; PDSQ: Psychiatric Diagnostic Screening Questionnaire; PHQ-9: Personal Health Questionnaire-9; PHX: Psychiatric history; PRISE: Patient Rated Inventory of Side Effect; PROMIS: Patient-Reported Outcomes Information System; PRS: Polygenic risk score; PSEQ: Pain Self-Efficacy Questionnaire; PSP: Personal and Social Performance; PSRs: Psychiatric Status Ratings; QIDS-C_16_: Quick Inventory of Depressive Symptomatology (Clinician-Rated); QIDS-SR_16_: Quick Inventory of Depressive Symptomatology (Self-assessment); QoL: Quality of life; RCT: randomized controlled trial; RF: Random Forest; rTMS: Repetitive transcranial magnetic stimulation; RMSE: Root mean squared error; RNN: Recurrent neural networks; SCAN: Schedules for Clinical Assessment in Neuropsychiatry; SCS: Suicide Cognitions Scale; SEWIP: Scale for the Multiperspective Assessment of General Change Mechanisms in Psychotherapy; SHAPS: Snaith Hamilton Pleasure Scale; SICD: Structured clinical interview for DSM-IV; sMRI: Structural Magnetic Resonance Imaging; SNPs: Single nucleotide polymorphism; SNRIs: Serotonin-norepinephrine reuptake inhibitors; SPE: Subjective Prognostic Employment Scale; SSRIs: Selective serotonin reuptake inhibitors; SVM: Support vector machine; TCAs: Tricyclic antidepressants; TNF: Tumor necrosis factor; TRD: Treatment-resistant depression; XGBoost: Extreme gradient boosting; YMRS: Young Mania Rating Scale; ω-3 PUFA: Omega-3 polyunsaturated fatty acids.

The most commonly used predictors included depression severity measures using different validated tools such as the Hamilton Depression Rating Scale (HDRS) (Athreya et al., 2021; Busk et al., 2020; Choo et al., 2024; Harrer et al., 2023; Wang, Wu, et al., 2024), Montgomerye-Åsberg Depression Rating Scale (MADRS) (Dong et al., 2024; Iniesta et al., 2016; Kautzky et al., 2018; Lee et al., 2021; Ricka et al., 2023), Beck Depression Inventory (BDI) (Choo et al., 2024; Furukawa et al., 2020; Hammelrath et al., 2024; Iniesta et al., 2016; Van Bronswijk et al., 2021), Personal Health Questionnaire-9 (PHQ-9) (Furukawa et al., 2020; Hammelrath et al., 2024; Harrer et al., 2023; Scodari et al., 2023), Hospital Anxiety and Depression Scale (HADS) (Scodari et al., 2023), and Quick Inventory of Depressive Symptoms, 16-item self-report version (QIDS-SR16) (Browning et al., 2019; Nie et al., 2018; Wang, Wu, et al., 2024). Demographic variables such as age, sex, body mass index (BMI), occupation, marital status, education level, and race were frequent predictors used in AI models (20/40) (Table 3). Medical history and comorbidities, including previous treatment experience, medication history, Axis II or III comorbidities, and concurrent physical illnesses, were also considered (15/20) (Table 3). Psychosocial factors such as stressful life events, socioeconomic status, quality of social, work, and family life were predictors used in several studies (13/40) (Table 3). General activity data such as physical activity, sleep and step data, phone usage (Barrigon et al., 2023; Ricka et al., 2023; Scodari et al., 2023; Zou et al., 2023), and physiological data including heart rate variability, acoustic variables, heart rate, and breathing rate are also utilized as predictors (Carreiro et al., 2024; Ricka et al., 2023; Wang, Wu, et al., 2024). Genetic factors, including single nucleotide polymorphisms (SNPs) and gene expression profiles, were examined in some studies (Guilloux et al., 2015; Maciukiewicz et al., 2018). Cognitive and neurobiological markers, such as electroencephalographic (EEG) measures, functional magnetic resonance imaging (fMRI) data, cognitive performance measures, and speech features, were utilized to assess cognitive functioning, neurobiological alterations, and affective processes (Bailey et al., 2018; Browning et al., 2019; Busk et al., 2020; Dong et al., 2024; Dougherty et al., 2023; Foster et al., 2019; Hilbert et al., 2024; Iniesta et al., 2016; Nguyen et al., 2022; Solomonov et al., 2021; Van Bronswijk et al., 2021; Weintraub et al., 2023). Finally, treatment-related variables, such as intervention assignment, treatment group, previous treatment response, and adherence, to pharmacotherapy were also included as predictors (Table 3).

### Intervention

Thirteen studies were included in the AI-assisted intervention, with 10 using the AI chatbot method (Danieli et al., 2022; Dimeff et al., 2021; Fulmer et al., 2018; Karkosz et al., 2024; Kleinau et al., 2024; Klos et al., 2021; Ogawa et al., 2022; Sabour et al., 2023; Schillings et al., 2023; Suharwardy et al., 2023). The remaining three studies involved using AI-based applications for medication reminders and drug identification (Chen et al., 2023), an AI platform aiding therapists in clinical decision-making and task automation (Sadeh-Sharvit et al., 2023), and an AI robotic puppy for interactive patient engagement (Yamada et al., 2024) (Table 4).

**Table 4.** Studies on AI-assisted interventions in mental health

Ref.	Subject description	Mental health condition	Aim	AI-based method	Intervention description	Control group intervention	Intervention duration	Outcome measures	Results and accuracy	Conclusions
Karkosz et al. (2024)	People reported at least mild depressive or anxiety symptoms (*n* = 81)	Depression, anxiety, positive and negative affect, global life satisfaction and loneliness	To assess Fido’s efficacy, a therapy chatbot targets depressive and anxiety symptoms through CBT techniques	AI chatbot	Fido focuses on dialogue to recognize and modify cognitive biases using Socratic questioning. It identifies suicidal ideation, guiding users to emergency hotlines. Fido utilizes the ABC technique from CBT, provides psychoeducation on mental health, and offers gratitude practice exercises	Received a book containing psychoeducation and self-help exercises, similar in content to that provided to the intervention group	Two weeks	CESD-R PHQ–9 PSWQ STAI PANAS SWLS Revised-UCLA Loneliness Scale	Depressive and anxiety symptoms decreased after the intervention and remained stable at the 1-month follow-up. Although loneliness was not significantly different between groups post-intervention, frequent Fido users showed a decline in loneliness	Fido provided sufficient help to reduce anxiety and depressive symptoms and decreased perceived loneliness among high-frequency users
Kleinau et al. (2024)	Health workers from public and private healthcare facilities (*n* = 1584)	Depression, anxiety, loneliness and burnout	To assess the effectiveness of the interactive chatbot, Vitalk, in improving mental well-being and resilience outcomes among health workers	AI chatbot	Vitalk utilizes an automated chatbot named Viki to provide mental health support through conversations based on CBT and positive psychology principles. Users engage in reflective discussions, access mood tracking tools, and participate in themed conversations to manage stress, mood, and anxiety. The platform also offers feedback, guidance, and emergency support information when necessary	Received access to a webpage with links to mental health resources (also wait list)	Eight weeks	PHQ–9 GAD–7 UCLA Loneliness Scale OLBI	Although there were statistically significant differences in the average scores for mental health (depression, anxiety, loneliness, and burnout) between the control and treatment groups, these differences were very small and both groups fell within the same risk category. The Difference-in-Differences estimates suggested a significant positive effect of Vitalk in reducing anxiety and depression. Depression showed the largest difference in effect size between the control and treatment groups	Vitalk’s positive impact on mental well-being and resilience makes it a promising tool against work-related stress and burnout
Schillings et al. (2023)	People without diagnosed mental disorders reported a moderate to high perceived stress level (PSS–10 score ≥ 14) (*n* = 118)	Stress and subjective well-being	To evaluate the effects of a chatbot intervention led by ELME on reducing stress and improving health-related parameters in individuals with medium to high stress levels	AI chatbot	ELME, a rule-based chatbot accessible as a web-based mobile application, provided psychoeducation, real-time dialogues, audio exercises, and personalized feedback to participants. It conducted two daily interactive intervention sessions (10–20 minutes each), focusing on stress, mindfulness, and interoception. The sessions were flexible, allowing participants to postpone exercises and receive SMS reminders	Usual care	Three weeks	PSS–4 WHO–5	There were no significant changes in perceived stress levels over time (from T1 to T3) and no significant effects of group or interactions between time and group on momentary perceived stress in the two models Subjective well-being showed an average improvement over time in both groups. No significant differences were found between the groups and there were no significant changes observed over time and between groups	Further research is needed to optimize the effectiveness of chatbot interventions for stress reduction through considerations such as intervention duration, target populations, and tailored approaches
Suharwardy et al. (2023)	Women within 72 hours postpartum (*n* = 192)	Depression and anxiety	To evaluate the acceptability and preliminary efficacy of a mental health chatbot for mood management in a general postpartum population	AI chatbot	Peripartum-specific content and psychotherapeutic techniques from CBT and IPT for postpartum mood were integrated into the AI chatbot to assist postpartum mothers in coping with mood and anxiety	Usual care	Six weeks	EPDS PHQ–9 GAD–7	There was a statistically significant difference in mean change scores from baseline to 6 weeks for PHQ–9 between the two groups. However, at the 6-week mark, there were no statistically or clinically significant variances between the groups in terms of EPDS scores, and there were also no differences observed in anxiety levels between the two groups	Given that the sample did not screen positive for depression at baseline, the potential of the chatbot to reduce depressive symptoms in this general obstetric population was limited
Yamada et al. (2024)	Patients with hematological malignancies who have undergone hematopoietic stem cell transplantation in a protective isolation unit (*n* = 21)	Stress and depressive symptoms	To examine if using a robotic puppy, aibo, could benefit the mental health of patients with hematological malignancies undergoing stem cell transplantation	AI robotic puppy with deep learning	Each patient was allowed to pet an aibo during the treatment period and was free to name the aibo Aibo uses sensing technology to recognize users and their surroundings, enabling it to assess situations and make decisions using artificial intelligence and deep learning. Its personality is shaped by interactions, experiences, and environmental mapping, allowing it to embody a unique animal-like character	Usual care	Entire stay in isolation unit	Salivary CgA Serum oxytocin Serum cortisol QIDS-J	At discharge, the intervention group exhibited a significant decrease in CgA, a significant increase in oxytocin, and a significantly more pronounced decrease in cortisol compared to the control group. Throughout the hospitalization period, the intervention group demonstrated a significant decrease in CgA levels and a significant increase in oxytocin levels, while the control group showed no significant change in CgA and a significant decrease in oxytocin No significant difference in the overall QIDS-J score, but there was a significant improvement in the psychomotor activity subscale in the intervention group	The AI robotic puppy intervention during a stay in an isolation unit can improve the mental health of patients with hematological malignancies
Chen et al. (2023)	Patients aged 20–65 with schizophrenia who lived at a psychiatric daycare center (*n* = 105)	Schizophrenia	To explore the effectiveness of intervention with the MedAdhere app on medication adherence and accuracy in patients with schizophrenia	AI-based app	Nighttime medication involves the MedAdhere app, a tool for medication management including scheduling, reminders, tracking, adherence assessments, and facial and antipsychotic recognition	Nighttime medication is self-administered by the patient without intervention	12 weeks	Medication Adherence Rate PANSS	Medication Adherence Rate IG: 94.72% CG: 64.43% Psychotic symptoms (positive, negative, and general psychopathology) significantly improved in the intervention group post-intervention compared to the control group	The app effectively and significantly improved medication adherence and the psychiatric symptoms of patients with schizophrenia
Sabour et al. (2023)	Healthy adults (*n* = 301)	Depression, anxiety, positive and negative affect	To evaluate Emohaa’s effectiveness in reducing mental distress symptoms through CBT-Bot exercises and guided conversations	AI chatbot	The CBT chatbot, rooted in CBT principles, uses interactive exercises like automatic thoughts training and guided expressive writing to address irrational thoughts and enhance mental well-being. Users engage in exercises via conversational choices, focusing on diary entries and hypothetical scenarios to gain new perspectives. Post-exercise, users report mood and emotions. The emotion support (ES) chatbot platform employs a strategy-driven dialogue model and a safety feature to detect suicidal signs, providing immediate help. It allows free-flowing conversations. Group 1: only CBT chatbot Group 2: CBT and ES chatbot	Wait list	Three weeks	PHQ–9 GAD–7 PANAS	Compared to the control group, participants who used two types of Emohaa experienced significantly more improvement in symptoms of mental distress, including depression and negative affect	Emohaa is a practical and effective tool for reducing mental distress
Sadeh-Sharvit et al., 2023	Participants diagnosed with depressive or anxiety disorders who require outpatient individual CBT (*n* = 47)	Depression and anxiety	To evaluate the feasibility, acceptability, and initial effectiveness of an AI platform designed to assist therapists in delivering mental health services	AI platform assists therapists in mental health services	The Eleos Health Platform is a secure tool for behavioral health professionals, supporting clinical decisions, automating tasks, analyzing therapist-patient dialogues, offering feedback on evidence-based practices, and facilitating measurement-based care and progress note generation. Therapists receive training to independently deliver interventions without prescribed practices	Usual care	Two months	PHQ–9 GAD–7	The intervention group attended 67% more sessions than the control group, with reductions of 34% in depression and 29% in anxiety, compared to 20% and 8% in the control group, respectively, showing significant benefits of therapy supported by the AI platform	Providing therapy in behavioral health settings with the support of an AI platform was more effective than usual care
Danieli et al. (2022)	Active workers over 55 with stress symptoms and mild-to-moderate anxiety (*n* = 60)	Stress and anxiety	To evaluate the contribution of TEO, a mobile personal health care agent with conversational AI	AI chatbot	TEO Mobile personal health care agent to recognize users’ emotional states, beliefs, and personal events, followed by implementing strategies designed by professionals	Traditional in-person therapy Mixed treatment with traditional in-person therapy and TEO mobile health agent No treatment	Eight weeks	SCL–90-R OSI PSS PHQ–8 GAD–7	In the mixed treatment (tradition in-person and TEO) group, statistically significant difference within group was observed in perceived stress, obsessiveness and compulsiveness, interpersonal sensitivity, depression, hostility, paranoid ideation, psychoticism, task-orientation, mental health, and physical health In the TEO group, statistically significant difference within group was observed in the interpersonal sensitivity and paranoid ideation In the tradition in-person group, statistically significant difference within group was observed in the paranoid ideation and logic In the no treatment group, no significant difference was observed	Mixed treatment with in-person and TEO components is the most effective in reducing stress and anxiety
Ogawa et al. (2022)	Patients aged 20 to 80 from the outpatient clinic with a diagnosis of clinically established or probable PD (*n* = 20)	Mood, mainly focusing on depression	To assess the feasibility and efficacy of using an AI-based chatbot to improve smile and speech in participants with PD, and to explore the potential predictive value of objective face and speech parameters for motor symptoms, cognition, and mood	AI chatbot	Daily chatbot (including multi-turn conversation to simulate a typical teleconsultation, with a report generated for each session) and weekly video-conferencing sessions with a neurologist	Weekly video-conferencing sessions with a neurologist.	Five months	BDI-II	A significant interaction effect was found on the smile index and speech features, but no significant interaction effects were observed for depression. The explorative analysis using statistical and machine learning models revealed that the smile indices and several speech features were associated with depression	An AI-based chatbot improves smile and speech in patients with PD, which indirectly capture the small improvement in depression that cannot be detected by conventional scales
Dimeff et al. (2021)	Individuals aged 18 or above who were suicidal and reached out to the Emergency department based psychiatric crisis services (*n* = 31)	Suicide	To evaluate the feasibility, acceptability, and effectiveness of a tablet-based app, Jaspr Health, among suicidal adults in Emergency Departments	AI tablet- based app with AI chatbot	Jaspr Health, a tablet-based app to conduct a comprehensive suicide risk assessment, a crisis stability plan, lethal means counseling, and education on behavioral skills to improve individual capacity to tolerate future crises, by identifying and treating patient- articulated drivers of suicide	Care as usual	Two hours	SIDQ SRCS	Significant decreases in distress and agitation, along with significant increases in learning to cope more effectively with current and future suicidal thoughts, were observed among participants using Jaspr Health compared with those receiving care as usual	Jaspr Health is feasible, acceptable, and clinically effective for use by patients at the Emergency Department who are suicidal and reach out to the Emergency Department based psychiatric crisis services
Klos et al. (2021)	University students aged 18 to 33 (*n* = 181)	Anxiety and depression symptoms	To evaluate the viability, acceptability, and potential impact of using Tess, an AI-based chatbot that delivers brief text conversations as comprehensive support for mental health	AI chatbot	A chatbot developed to send reminders, psycho- educational content, and emotional support responses based on what the users express	An electronic psychoeducation book on depression.	Eight weeks	PHQ–9 GAD–7	The GAD–7 score of the participants in the AI chatbot group was significantly reduced compared to the control group No significant difference in the PHQ–9 score was observed	Tess is effective in addressing anxiety but not depressive symptoms
Fulmer et al. (2018)	Students aged 18 and older (n = 75)	Anxiety and depression symptom	To assess the feasibility and efficacy of using an integrative psychological AI, Tess, to reduce self-identified symptoms of depression and anxiety in college students	AI chatbot	A psychological AI chatbot designed to deliver brief conversations in the form of integrative mental health support, psychoeducation, and reminders	An electronic link to the National Institute of Mental Health’s eBook on depression among college students	Two or four weeks	PHQ–9 GAD–7 PANAS	A significant difference was found on the PHQ–9 and the PANAS between participants in the AI chatbot and the control group after a two-week use of AI chatbot. The score of GAD–7 of the participants with two-week or four-week use of AI chatbot was significantly reduced compared to the control group	Psychological AI has the potential to reduce symptoms of depression and anxiety by delivering CBT-based interventions in the form of conversations

Abbreviations: AI: Artificial Intelligence; BARS: Brief Agitation Rating Scale; BDI-II: Beck Depression Inventory-II; CBT: Cognitive Behavioral Therapy; CESD-R: Center for Epidemiologic Studies Depression Scale Revised; CgA: Chromogranin A; CSDD: Cornell Scale for Symptoms of Depression in Dementia; EPDS: Edinburgh Postnatal Depression Scale; GAD-7: General Anxiety Disorders-7 scales; OLBI: Oldenburg Burnout Inventory; OSI: Occupational Stress Indicator; PANAS: Positive and Negative Affect Scale; PANSS: Positive and Negative Syndrome Scale; PD: Parkinson’s Disease; PHQ-8: Patient Health Questionnaire-8; PHQ-9: Patient Health Questionnaire-9; PSS: Perceived Stress Scale; PSWQ: Penn State Worry Questionnaire; QIDS: Quick Inventory of Depressive Symptomatology Self-Report; SCL-90-R: Symptom Checklist-90-Revised; SIDQ: Safety and Imminent Distress Questionnaire; SRCS: Suicide-Related Coping Scale; STAI: State–Trait Anxiety Inventory; SWLS: Satisfaction With Life Scale; TEO: Therapy Empowerment Opportunity; WHO-5: 5-item WHO Well-Being Index.

The studies compared the treatment effectiveness of AI-assisted interventions against traditional interventions (Danieli et al., 2022; Dimeff et al., 2021; Ogawa et al., 2022; Sadeh-Sharvit et al., 2023; Schillings et al., 2023; Suharwardy et al., 2023; Yamada et al., 2024) or psychoeducation (Fulmer et al., 2018; Karkosz et al., 2024; Kleinau et al., 2024; Klos et al., 2021), and other studies compared mixed treatment and no treatment (Chen et al., 2023; Danieli et al., 2022; Sabour et al., 2023). Subjects were adults with depressive, anxiety, schizophrenia, stress, and/or suicidal symptoms, with or without an established diagnosis, with a total sample size of 2816. The most prevalent mental health conditions treated with AI-assisted inventions were depression and anxiety. PHQ-8 or -9 and GAD-7 were common outcome measures evaluated in AI-assisted intervention studies (Table 4).

### Quality assessment

The quality of the studies was assessed using the NIH assessment tools. Fifty studies were rated as good, 34 studies as fair, and one study as poor (Table 5). Within the diagnosis domain, there was one controlled intervention study, 15 observational cohort and cross-sectional studies, and 16 case-control studies, 18 rated as good, 13 as fair, and one as poor. One article falls under both the diagnosis and monitoring domains, classified as observational cohort and cross-sectional studies, and assessed as fair. Regarding the intervention domain, all 13 studies were controlled intervention studies, with five rated as good and eight as fair (Table 5).

**Table 5.** Result of the individual components of the quality assessment of the included studies

Domain	References	Rating	1. Described as randomized	2. Randomized treatment assignment	3. Concealed treatment allocation	4. Blinding of treatment group assignment	5. Assessor’s blinding	6. Similarity of groups at baseline	7. Overall drop-out	8. Between groups drop-out	9. Adherence	10. Avoid other interventions	11. Outcome measures assessment	12. Power calculation	13. Prespecified outcomes	14. Intention-to-treat analysis
Quality assessment of controlled intervention studies																
Diagnosis	Jaroszewski et al. (2019)	Fair	Y	Y	Y	Y	Y	Y	N	Y	N	Y	Y	N	Y	Y
Monitoring	Dong et al. (2024)	Good	Y	Y	Y	Y	Y	Y	NR	NR	NR	Y	Y	Y	Y	Y
	Wang, Wu, et al. (2024)	Fair	Y	Y	NR	NR	NR	Y	Y	Y	Y	NR	Y	NR	Y	NR
	Wang, Wu, et al. (2024)	Fair	Y	NA	NA	NR	NR	Y	NR	NR	NR	NR	Y	Y	Y	NR
	Zainal and Newman (2024)	Good	Y	Y	Y	Y	Y	Y	Y	Y	NR	Y	Y	Y	Y	Y
	Brandt et al. (2023)	Fair	Y	NA	NA	NR	NR	Y	Y	N	NA	NA	Y	NA	Y	NA
	Dougherty et al. (2023)	Good	Y	Y	Y	Y	Y	Y	Y	Y	NR	N	Y	Y	Y	Y
	Harrer et al. (2023)	Fair	Y	Y	N	N	Y	Y	N	N	Y	N	Y	Y	Y	Y
	Scodari et al. (2023)	Good	Y	Y	N	N	Y	Y	Y	N	Y	N	Y	Y	Y	Y
	Webb et al. (2022)	Good	Y	Y	Y	NR	Y	Y	N	N	Y	N	Y	Y	Y	Y
	Lee et al. (2021)	Good	Y	Y	Y	N	Y	Y	Y	NR	Y	N	Y	N	Y	Y
	Van Bronswijk et al. (2021)	Good	Y	Y	Y	Y	Y	Y	Y	Y	Y	N	Y	Y	Y	Y
	Busk et al. (2020)	Good	Y	Y	Y	NR	Y	Y	N	Y	Y	Y	Y	N	Y	NR
	Rajpurkar et al. (2020)	Good	Y	Y	Y	Y	Y	Y	Y	Y	Y	N	Y	Y	Y	Y
	Rozek et al. (2020)	Fair	Y	Y	Y	Y	Y	Y	Y	Y	NR	Y	Y	N	Y	Y
	Foster et al. (2019)	Good	Y	Y	Y	Y	Y	Y	N	Y	N	Y	Y	N	Y	Y
	Vitinius et al. (2019)	Fair	Y	Y	Y	Y	Y	Y	NR	NR	NR	Y	Y	N	Y	NR
	Kautzky et al. (2018)	Good	Y	Y	Y	Y	Y	Y	Y	Y	Y	N	Y	Y	Y	Y
	Amminger et al. (2015)	Good	Y	Y	Y	N	Y	Y	NR	NR	NR	Y	Y	N	Y	Y
Intervention	Karkosz et al. (2024)	Fair	Y	Y	Y	N	NR	Y	Y	Y	Y	N	Y	N	Y	Y
	Kleinau et al. (2024)	Good	Y	Y	Y	Y	Y	Y	N	Y	Y	N	Y	Y	Y	Y
	Schillings et al. (2023)	Fair	Y	Y	N	N	N	Y	N	N	N	N	Y	Y	Y	Y
	Suharwardy et al. (2023)	Fair	Y	Y	N	N	N	Y	N	N	Y	N	Y	Y	Y	NR
	Yamada et al. (2024)	Fair	Y	Y	N	N	NR	Y	Y	Y	NR	N	Y	N	NR	NR
	Chen et al. (2023)	Good	Y	Y	Y	N	Y	Y	Y	Y	Y	N	Y	Y	Y	NR
	Sabour et al. (2023)	Fair	Y	Y	N	NR	NR	Y	N	NR	Y	NR	Y	Y	Y	Y
	Sadeh-Sharvit et al. (2023)	Fair	Y	Y	N	N	N	Y	Y	Y	Y	N	Y	N	Y	Y
	Danieli et al. (2022)	Good	Y	Y	N	N	N	Y	N	Y	NA	N	Y	N	Y	Y
	Ogawa et al. (2022)	Fair	Y	Y	Y	NR	NR	Y	Y	Y	NR	Y	Y	N	Y	Y
	Dimeff et al. (2021)	Good	Y	Y	N	NR	NR	Y	Y	NA	Y	N	Y	N	Y	Y
	Klos et al. (2021)	Good	Y	Y	Y	NA	Y	Y	Y	Y	Y	NR	Y	Y	Y	NR
	Fulmer et al. (2018)	Fair	Y	Y	N	NR	NR	Y	Y	Y	NR	N	Y	N	Y	Y
Quality assessment tool for observational cohort and cross-sectional studies																
Diagnosis	Yang et al. (2024)	Fair	Y	Y	NR	Y	N	Y	Y	NR	Y	Y	Y	NR	NR	NR
	Kourou et al. (2023)	Fair	Y	Y	NR	Y	N	Y	Y	NR	Y	Y	Y	NR	NR	NR
	Lønfeldt et al. (2023)	Fair	Y	Y	NR	Y	Y	Y	Y	NR	N	Y	Y	NR	Y	NR
	C Manikis et al. (2023)	Fair	Y	Y	NR	Y	N	Y	Y	NR	Y	Y	Y	NR	NR	N
	Adler et al. (2022)	Fair	Y	Y	NR	Y	N	Y	Y	Y	Y	Y	N	NR	NR	NR
	Hüfner et al. (2022)	Good	Y	Y	NR	Y	N	Y	Y	Y	Y	Y	Y	NR	Y	Y
	Matsuo et al. (2022)	Good	Y	Y	NA	Y	Y	Y	Y	Y	Y	N	Y	NR	NA	N
	Susai et al. (2022)	Good	Y	Y	Y	Y	N	Y	Y	Y	Y	Y	Y	NR	Y	Y
	Andersson et al. (2021)	Good	Y	Y	NR	Y	N	Y	Y	N	Y	Y	Y	NR	Y	Y
	Du et al. (2021)	Poor	Y	N	NR	CD	N	Y	NR	NR	N	N	N	NR	NA	NR
	Tate et al. (2020)	Good	Y	Y	Y	Y	N	Y	Y	Y	N	N	Y	NR	NA	Y
	Simon et al. (2019)	Good	Y	Y	Y	Y	N	Y	Y	N	Y	Y	Y	NR	NR	Y
	Xu et al. (2018)	Good	Y	Y	NR	Y	N	Y	Y	Y	Y	N	Y	NR	NA	Y
	Cook et al. (2016)	Fair	Y	Y	NR	Y	N	Y	Y	N	Y	Y	Y	NR	NR	N
	Setoyama et al. (2016)	Fair	Y	Y	NR	N	N	Y	NR	Y	Y	N	Y	NR	NR	Y
Monitoring	Carreiro et al. (2024)	Fair	Y	Y	Y	Y	N	Y	Y	NR	Y	Y	N	NR	NA	NR
	Choo et al. (2024)	Good	Y	Y	Y	Y	Y	Y	Y	NR	Y	Y	Y	NR	NR	NR
	Barrigon et al. (2023)	Fair	Y	Y	Y	Y	N	Y	Y	NR	Y	Y	N	NR	NR	N
	Ricka et al. (2023)	Good	Y	Y	Y	Y	N	Y	Y	NR	Y	Y	Y	NR	NR	NR
	Zou et al. (2023)	Good	Y	Y	Y	Y	N	Y	Y	NR	Y	Y	Y	NR	N	NR
	Weintraub et al. (2023)	Fair	Y	Y	Y	Y	N	Y	Y	N	N	Y	Y	NR	Y	Y
	Athreya et al. (2021)	Fair	Y	Y	NR	Y	N	Y	Y	NR	Y	Y	Y	NR	NR	Y
	Solomonov et al. (2021)	Fair	Y	Y	NR	Y	N	Y	Y	N	Y	Y	Y	N	NR	Y
	Bailey et al. (2018)	Good	Y	Y	NR	N	N	Y	Y	Y	Y	Y	Y	NR	N	NR
	Kautzky et al. (2018)	Good	Y	Y	Y	Y	NR	Y	Y	Y	Y	N	Y	N	N	Y
	Maciukiewicz et al. (2018)	Good	Y	N	N	Y	N	Y	Y	NR	Y	Y	Y	NR	NR	Y
	Chekroud et al. (2016)	Good	Y	Y	Y	Y	N	Y	Y	NR	Y	Y	Y	NR	Y	N
Diagnosis and monitoring	Jacobson et al. (2022)	Fair	Y	N	Y	Y	N	N	Y	Y	Y	N	Y	NR	NA	N

Domain	References	Rating	1. Clearly stated research question	2. Clearly defined population	3. Sample size justification	4. Controls recruited from similar population	5. Prespecified eligibility uniformly applied	6. Case & controls differentiation	7. Random selection of study participants	8. Concurrent controls	9. Exposure assessed prior to outcome measurement	10. Exposure measures & assessment	11. Blinding of exposure assessors	12. Adjusted key potential confounding variables		
Quality assessment of case-control studies																
Diagnosis	Chen et al. (2024)	Good	Y	Y	N	Y	Y	Y	NR	Y	Y	Y	NR	Y		
	Das and Naskar (2024)	Fair	Y	Y	N	N	N	Y	NR	Y	Y	Y	NR	NR		
	Maekawa et al. (2024)	Good	Y	Y	N	Y	N	Y	Y	Y	Y	Y	NR	NR		
	Geng et al. (2023)	Fair	Y	Y	N	NR	N	Y	Y	Y	Y	N	NR	NR		
	Chilla et al. (2022)	Fair	Y	Y	N	N	Y	Y	NA	Y	NR	NR	NR	Y		
	Mongan et al. (2021)	Good	Y	Y	N	N	Y	Y	Y	Y	Y	Y	N	Y		
	Tsui et al. (2021)	Good	Y	Y	Y	N	Y	Y	Y	N	Y	Y	Y	Y		
	Maglanoc et al. (2020)	Fair	Y	Y	N	N	Y	Y	NR	NR	NR	NR	NR	Y		
	Byun et al. (2019)	Good	Y	Y	Y	Y	Y	Y	NR	Y	Y	Y	NR	Y		
	Ebdrup et al. (2019)	Good	Y	Y	N	Y	Y	Y	NR	Y	N	Y	NR	Y		
	Lyu and Zhang (2019)	Good	Y	Y	Y	Y	Y	Y	Y	Y	Y	Y	NR	Y		
	Carrillo et al. (2018)	Fair	Y	N	N	Y	N	Y	Y	Y	Y	Y	NR	NR		
	Liang et al. (2018)	Good	Y	Y	N	Y	N	Y	NA	Y	NR	NR	NR	Y		
	Pestian et al. (2016)	Good	Y	Y	N	Y	Y	Y	NR	N	N	NR	NR	Y		
	Schnack et al. (2014)	Good	Y	Y	Y	N	Y	Y	Y	Y	Y	Y	NR	Y		
	Marquand, Mourão-Miranda, Brammer, Cleare, & Fu, (2008)	Good	Y	Y	N	Y	Y	Y	NR	Y	Y	Y	NR	N		
Monitoring	Nie et al. (2018)	Good	Y	Y	N	Y	Y	Y	Y	Y	Y	Y	NR	Y		
	Guilloux et al. (2015)	Good	Y	Y	N	Y	Y	Y	Y	Y	Y	Y	NR	Y		
Quality assessment of before-after (pre–post) studies with no control group																
Monitoring	Hammelrath et al. (2024)	Good	Y	Y	Y	Y	Y	Y	Y	NR	N	Y	N	Y		
	Hilbert et al. (2024)	Good	Y	Y	Y	Y	Y	Y	Y	NR	N	Y	N	NR		
	Jankowsky et al. (2024)	Good	Y	Y	Y	Y	Y	Y	Y	NR	N	Y	N	NR		
	Bao et al. (2021)	Fair	Y	N	Y	Y	N	Y	Y	NR	N	N	N	Y		
	Furukawa et al. (2020)	Good	Y	Y	Y	Y	Y	Y	Y	Y	Y	Y	N	NA		
	Browning et al. (2019))	Good	Y	Y	Y	Y	N	Y	Y	NR	Y	Y	Y	Y		
	Iniesta et al. (2016)	Good	Y	Y	Y	Y	Y	Y	Y	NR	N	Y	N	Y		

Abbreviations: Y: yes; N: no; NA: not applicable; NR: not reported.

## Discussion

Among the 85 articles included, 58.8% were rated as good, and 40% were rated as fair. Within the monitoring domain, 69% of the articles were rated as good, while in the diagnosis domain, 56% were rated as good. In the intervention domain, 38% of the articles were rated as good. In controlled intervention studies, the main factors impacting the quality of the articles include the absence of reporting adherence and drop-out rates, as well as insufficient description and implementation of concealment and blinding methods, particularly within the intervention domain. For observational cohort and cross-sectional studies, the main factors impacting quality were the lack of reporting or insufficient information regarding the participation rate, follow-up loss, blinding, sample size justification, and adjustment for key potential confounding variables. In case-control studies, the quality was primarily affected by the absence of reporting or insufficient information on sample size justification, random selection of study participants, and blinding of exposure assessors. In pre–post studies with no control group, the quality was significantly influenced by the lack of information on blinded outcome assessors, follow-up loss, and failure to utilize an interrupted time-series design. These issues stem from the fact that some AI models are trained on existing datasets, which are not always original data and sometimes involve the use of multiple datasets for training, making it challenging to adapt to evaluation frameworks. The overall quality of the studies is good, with 58.8% rated positively, which strengthens the review’s conclusions. However, deficiencies in reporting and methodology, especially in intervention studies where only 38% were rated as good, warrant caution in interpreting the results due to potential biases and limitations.

Studies of machine learning, within the diagnosis domain, demonstrated varying performances in detecting, classifying, and predicting the risk of having a mental health condition. Up to 28 studies reported accuracy in classifying or predicting mental health conditions, ranging from 51% to 97.54% (Table 2). Machine learning models based on a single predictor, such as heart rate variability features (Byun et al., 2019), EEG signals (Du et al., 2021), MRI data (Marquand et al., 2008), audio spectrogram (Das & Naskar, 2024), or gray matter density (Schnack et al., 2014) already accomplished satisfactory performance with reported accuracies ranging from 68% to 97.54%. Surprisingly, increasing the number of predictors did not increase the predictive power or classification performance of the machine model concerned (Andersson et al., 2021; Ebdrup et al., 2019; Yang et al., 2024). Designing and selecting different models and variables for prediction can lead to varying outcomes when applied to the same population with different baselines (Manikis et al., 2023). Yang et al. (2024) discovered that notable differences were evident when considering 10 to 15 variables across various variable transformation methods. They found that using more than 15 variables in the model did not significantly improve accuracy. Furthermore, as the number of included variables increases, the practical complexity also rises. Given these conclusions and findings, the significance of targeted variable selection is underscored and warrants further exploration. In general, machine learning demonstrated satisfactory to good performance (accuracy level above 75%) in detecting, classifying, and predicting the risk of having a mental health condition.

Support vector machine is a machine learning model often used for diagnosing mental health conditions, employing a linear decision boundary, or ‘hyperplane’, to effectively separate classes in a dataset (Mohamed et al., 2023). It has shown high accuracy in diagnosing anxiety (95%) and depression (95.8%) while achieving lower accuracy for bipolar disorder (69%) and PTSD (69%) among war veterans (Chung & Teo, 2022). Compared to random forest, which yields slightly lower accuracy rates (e.g., 78.6% for depression and anxiety), support vector machine is preferred for its ability to classify both linear and nonlinear data through kernel functions, despite its sensitivity to kernel choice and performance challenges with large or noisy datasets (Chung & Teo, 2022; Mohamed et al., 2023). Random forest, a supervised learning technique that combines multiple decision trees via bagging, offers improved accuracy and reduces overfitting but comes with increased computational complexity and limited interpretability (Mohamed et al., 2023). Both models face challenges like small sample sizes and inadequate validation that mental health care providers and researchers should be aware of, underscoring the need for high-quality data and more explainable models in mental health research (Chung & Teo, 2022).

Four limitations were identified for the use of AI to diagnose mental health conditions. First, the design of some AI-based diagnostic tools may be biased. Tate et al. (2020) described that the AI model used in their study may have two types of bias: reporting bias, as the outcome and the most important variables were all parent-reported, and bias in the variable importance with the use of mixed data types. Maekawa et al. (2024), Kourou et al. (2023), and Jaroszewski et al. (2019) argued that self-reported variables exhibit excessive subjectivity, and Kourou et al. (2023) further suggested that although they included a substantial number of variables in their study, the coverage remains insufficient, all of which may lead to biases. Matsuo et al. (2022) and Chen et al. (2024) reported that the incorporation of insufficient variables and an imbalanced dataset in developing the AI model may lead to bias. Jacobson et al. (2022) mentioned that there may be interrater bias regarding the features provided by their online mental health screening tools. Byun et al. (2019) stated that the classification algorithms were less accurate for high-dimensional data. Setoyama et al. (2016) mentioned that confounding variables influenced the results of the prediction model. Second, due to confounding variables and specific populations, AI models might reveal correlations between mental health conditions and other variables, yet they are unable to establish causality (Maekawa et al., 2024; Simon et al., 2019; Xu et al., 2018). Third, the application of AI-assisted diagnosis included trade-offs between different performance metrics, for instance, between model specificity and sensitivity (Adler et al., 2022; Andersson et al., 2021; Chilla et al., 2022; Cook et al., 2016). Fourth, in addition to the constraints mentioned above, including the prevalent use of singular datasets and small sample sizes in studies, as well as technical issues, the AI-assisted diagnostic tools model exhibited limited generalizability (Chen et al., 2024; Geng et al., 2023; Kourou et al., 2023; Lønfeldt et al., 2023; Maekawa et al., 2024). Liang et al. (2018) and Kourou et al. (2023) acknowledged that their model needed to be validated in other contexts. The above limitations should be considered for the optimal development and higher accuracy of AI-assisted diagnostic tools.

The application of AI to diagnose mental health conditions has brought several challenges. One of these was related to the ability to organize or generalize mental health conditions, major variables in developing the AI model, or standardized measures for the AI-assisted diagnosis. Maglanoc et al. (2020) mentioned that the nature of mental disorders was clinically highly heterogeneous and thus might not have the convergence for stratification to develop the AI models. Schnack et al. (2014) suggested that interpreting the effects of specific brain regions with AI was complicated since the discriminative brain pattern was a description of the cumulative contributions of all features. According to Adler et al. (2022), developing standardized measures of in-the-moment symptoms for continuous remote symptom assessment studies was challenging as it was difficult to align outcome symptom measures across studies for model development. Challenges of AI applied in diagnosis at the model-specific level were also identified. For instance, the design of the convolutional neural network (CNN) model required careful setup adjustment to accommodate input size and training objectives, including the network depth, the number of function mappings, and the kernels for each layer (Du et al., 2021). Cross-cultural variations and real-world resource constraints pose challenges for implementing clinical recommendations derived from AI models.

Six studies discussed ethical considerations surrounding the application of AI in diagnosing mental health issues (Adler et al., 2022; Jacobson et al., 2022; Jaroszewski et al., 2019; Lønfeldt et al., 2023; Maekawa et al., 2024; Tsui et al., 2021). The primary concern regarding AI models is focused on safeguarding privacy, with all included papers in agreement on the necessity of obtaining informed consent from data sources or patients. Adler et al. (2022), Jacobson et al. (2022), and Jaroszewski et al. (2019) also concurred that personally identifiable information should not be recorded or extracted. In addition to using privacy and data protection technologies, it is essential to offer appropriate knowledge support to subjects to address their concerns (Lønfeldt et al., 2023). Another ethical consideration involves providing assistance to high-risk participants. Tsui et al. (2021) suggested that clinicians and patients should be informed about risk data and potential treatment options. However, it is essential to remember that when using AI for diagnostic purposes, respecting a patients’ right to provide informed consent is crucial to prevent data misuse and misinterpretation. Additionally, it is important to avoid overestimating the efficacy of AI models, as this could introduce biases and risks. The challenge of balancing privacy protection when aiding high-risk individuals (e.g., suicidal ideation) remains unresolved. Researchers must proceed with caution, ensuring the legal and ethical utilization of data, even when readily available (Maekawa et al., 2024).

In the monitoring domain, most studies have explored the use of predictive models for treatment response in different psychiatric disorders, particularly depression. In terms of performance, AI has provided a variety of algorithms or models, showing promising prospects. Chekroud et al. (2016) demonstrated that machine learning achieved moderate performance in predicting treatment outcomes in different treatment groups. Lenhard et al. (2018) reported that machine learning models had good to excellent accuracy in predicting treatment outcomes in internet-delivered CBT for pediatric obsessive-compulsive disorder. Maciukiewicz et al. (2018) indicated that machine learning models had moderate performance in predicting treatment response but were less successful in predicting remission. Bailey et al. (2018) investigated baseline and week one measures of theta power and connectivity, which showed potential for predicting response to repetitive transcranial magnetic stimulation (rTMS) treatment. Kautzky et al. (2018) examined machine learning techniques and found promising results in predicting treatment-resistant depression. Foster et al. (2019) showed that treatment combined with CBT and fluoxetine consistently outperformed either therapy alone. Furukawa et al. (2020) suggested the use of support vector machines to predict treatment outcomes in different treatment arms. Athreya et al. (2021) identified four depressive symptoms and specific thresholds of change that predicted treatment outcomes with an average accuracy of 77%. Bao et al. (2021) employed machine learning models using genotyping information to predict treatment outcomes of ketamine infusions. Nguyen et al. (2022) demonstrated that predictive models could offer a possible precision medicine approach for antidepressant selection. Dong et al. (2024) proposed that a sequential modeling approach enhances the predictive responsiveness of patients with schizophrenia to rTMS treatment while simultaneously reducing diagnostic complexity. Wang, Wu, et al. (2024) demonstrated that the machine learning pipeline exhibited high accuracy and area under the receiver operating characteristic curve (AUC) (>0.80) on the training set when predicting treatment responses for patients with major depressive disorder using neuroimaging data, although extensive external validation is required.

These studies have involved a variety of treatment responses, including medication, psychology, and care. The predictive factors for these responses range from basic sociodemographic characteristics and treatment-related variables to genomics, acoustics, and other biomarkers. Amminger et al. (2015) conducted univariate and multivariate analyses, finding that fatty acids and symptoms could predict functional improvement in both the Omega-3 polyunsaturated fatty acids (ω-3 PUFA) and placebo groups. Guilloux et al. (2015) found that gene expression profiles obtained from blood samples could predict remission and nonremission outcomes in response to citalopram treatment for depression. Iniesta et al. (2016) discovered that demographic and clinical variables could predict therapeutic response to escitalopram with clinically significant accuracy. Nie et al. (2018) suggested that machine learning models using clinical and sociodemographic data could predict treatment-resistant depression. Browning et al. (2019) found that cognitive and symptomatic measures were useful in guiding antidepressant treatment. Rajpurkar et al. (2020) identified certain symptoms that exhibited high discriminative performance in predicting treatment outcomes, with baseline symptom severity being a critical predictor. Busk et al. (2020) found that historical mood was the most important predictor of future mood and that different mood scores exhibit correlation. Jacobson et al. (2022) found that online screening for depression influenced help-seeking behavior, suicidal ideation, suicidal intent, and identified individuals who may benefit from treatment interventions. Dougherty et al. (2023) suggested that treatment response for patients with treatment-resistant depression to psilocybin can be accurately predicted using a logistic regression model that incorporates Emotional Breakthrough Index metrics, natural language processing metrics, and treatment arm data. Harrer et al. (2023) found that a multivariate tree learning model predicts that patients with lower back pain and moderate depression, coupled with relatively low pain self-efficacy, benefit the most from an internet-based depression intervention. Jankowsky et al. (2024) highlighted that treatment-related variables play a pivotal role in predicting treatment response in naturalistic inpatient samples with anxious and depressive symptoms. Scodari et al. (2023) discovered that patients with depressive symptoms who underwent stepped care were more likely to reduce PHQ-9 scores if they had high PHQ-9 but low HADS-Anxiety scores at baseline, a low number of chronic illnesses, and an internal locus of control. Wang, Wu, et al. (2024) suggested that speech features, particularly energy parameters, serve as precise and objective indicators for tracking biofeedback therapy response and predicting efficacy for college students with symptoms of anxiety or depression. Hammelrath et al. (2024) emphasized that therapeutic alliance and early symptom change are crucial predictors for anticipating non-response to a 6-week online depression program. Zainal and Newman (2024) identified predictors, such as higher anxiety severity, elevated trait perseverative cognition, lower set-shifting deficits, older age, and stronger trait mindfulness, for individuals with generalized anxiety disorder who benefit from mindfulness ecological momentary intervention.

Using AI in predicting treatment response or prognosis of mental health disorders has limitations related to data availability, model performance, and external validity. First, data quality, cost, and sample size can affect the performance of AI models (Athreya et al., 2021; Bao et al., 2021; Brandt et al., 2023; Dougherty et al., 2023; Maciukiewicz et al., 2018; Wang, Wu, et al., 2024). Second, overfitting is a common problem, and the trade-off between data quality and model robustness can affect model performance (Bailey et al., 2018; Busk et al., 2020; Scodari et al., 2023; Susai et al., 2022). Third, AI models built on diverse populations and interventions, selecting a variety of diverse predictor variables, not only make it challenging to compare or replicate across different datasets, limiting the assessment of specific predictive factors, but may also fail to generalize to new samples, treatment settings, and populations (Brandt et al., 2023; Chekroud et al., 2016; Choo et al., 2024; Dong et al., 2024; Dougherty et al., 2023; Rajpurkar et al., 2020; Ricka et al., 2023). Researchers should take steps to mitigate these limitations, such as using standardized experimental protocols and platforms, collecting complete data over an extended period, and testing the generalizability of AI models in routine clinical settings (Busk et al., 2020; Foster et al., 2019).

Several challenges were identified in developing and applying treatment outcome prediction models. Browning et al. (2019) noted the difficulty in predicting remission of a mental health condition when the condition was less common. Busk et al. (2020) identified the challenge of collecting complete histories over a longer time for a better prediction model, and the challenge of developing a real-time forecast system due to the intervention, which can potentially change the outcome and future training data. Chekroud et al. (2016) pointed out identification difficulties regarding the variables to be used in the prediction model. Choo et al. (2024) emphasized that AI models may lack transparency regarding how input features influence predictions, thereby complicating assessments of predictor importance and causal inference. This presents a dual challenge for bias analysis and ethical considerations. Additionally, Guilloux et al. (2015) mentioned the challenge of directly applying predictive models to different test studies due to cross-laboratory variability in probe designs from different experimental protocols and different array platforms. These interplatform differences underscore the complexity of real-world scenarios, necessitating larger sample sizes and multicenter experiments in future research. However, this approach also brings about heightened risks of data leakage (Hilbert et al., 2024). These challenges highlight the importance of continued research and maintaining ethical integrity to improve the accuracy and generalizability of outcome prediction models.

Ethical considerations relating to the use of AI for monitoring mental health and predicting treatment response or prognosis of mental health disorders share key points with AI for diagnosis, emphasizing the critical importance of safeguarding patient privacy, informed consent, and autonomy. Informed consent stands as a fundamental element in these domains. One ethical concern was related to the data collected from electronic devices such as smartphones. These data should be stored on a secure server to ensure confidentiality and protect the participants’ privacy (Busk et al., 2020). Furthermore, the protocol for using AI in mental health should be approved by the ethics boards of all centers involved to ensure the safety and privacy of the participants (Iniesta et al., 2016). Using AI for monitoring can be highly beneficial for patients, especially those at high risk, such as individuals prone to suicide (Barrigon et al., 2023; Choo et al., 2024). However, implementing this while ensuring patient privacy is maintained is a crucial element that future ethical considerations must address. Simultaneously, researchers must be mindful of the opacity of AI and the potential for bias, exercising caution against overly exaggerating the capabilities of AI (Choo et al., 2024).

AI chatbots were used to investigate the effectiveness of AI-assisted treatment (Danieli et al., 2022; Dimeff et al., 2021; Fulmer et al., 2018; Karkosz et al., 2024; Kleinau et al., 2024; Klos et al., 2021; Ogawa et al., 2022; Sabour et al., 2023; Schillings et al., 2023; Suharwardy et al., 2023). AI chatbots showed inconsistent performance in treating mental health conditions. Dimeff et al. (2021) and Fulmer et al. (2018) found that AI chatbots contributed to significant improvements in reducing suicidal, depressive, or anxiety symptoms. Sabour et al. (2023) observed significantly greater improvement in symptoms of depression and negative affect with the chatbot. Karkosz et al. (2024) found that the chatbot reduced anxiety and depressive symptoms and decreased perceived loneliness among high-frequency users. Kleinau et al. (2024) reported a significant positive effect of Vitalk in reducing anxiety and depression.

Ogawa et al. (2022) showed that their AI chatbot only made small improvements in depression. Klos et al. (2021) indicated that their AI chatbot was only effective for anxiety but not for depressive symptoms. Danieli et al. (2022) concluded that a combination of in-person and AI treatment was more effective in reducing stress and anxiety than an AI chatbot alone. Schillings et al. (2023) did not find the chatbot to be more effective than usual care in reducing stress and enhancing subjective well-being. Suharwardy et al. (2023) concluded that the potential of the chatbot to reduce depressive symptoms in the general obstetric population was limited. AI chatbots generally use natural language processing techniques to understand and reply to questions from humans (Lalwani et al., 2018). In recent years, there has been a rise of generative AI chatbots, such as ChatGPT by OpenAI, which use transformer neural networks and large-scale language models (Atallah et al., 2023; Jo et al., 2023). These generative AI chatbots are now being tested and used in various application domains, such as the service industry, the creative industry, banking and finance, and even healthcare. However, further investigation is required to understand the use of these generative AI chatbots for the intervention of mental health disorders. Apart from chatbots, AI has shown significant potential in various applications within the medical field. Three additional studies employed AI for intervention assistance: Chen et al. (2023) used AI for medication reminders and identification, resulting in significantly improved medication adherence. Sadeh-Sharvit et al. (2023) used AI to aid therapists in mental health services, leading to increased patient session attendance. Yamada et al. (2024) introduced an AI puppy into a protective isolation unit, which notably reduced patients’ CgA and cortisol levels while increasing oxytocin production.

In addition, machine learning was found to be effective both in terms of treatment modalities and frequency recommendations for depression. Bruijniks et al. (2022) showed that stratified care with a machine learning model was efficacious for treatment selection. Delgadillo et al. (2022) reported that machine learning enhanced recommendations for a minority of participants. Furukawa et al. (2020) indicated that machine learning was able to predict the optimal frequency of CBT sessions. The basic approach in these studies is to first collect patient data, identify the predictive features, and then build the machine learning model that can predict the treatment modalities and frequency recommendations. These studies often leverage simple yet interpretable regression and classification models, including linear regression, logistic regression, decision trees, and support vector machines, instead of complex neural network models (Bruijniks et al., 2022; Furukawa et al., 2020).

There are some limitations to the use of AI in mental health interventions, as mentioned by the authors of some of the studies included. Fulmer et al. (2018) summarized the following four limitations of AI chatbots: (1) emotional identification was found to be limited to language and the chatbots did not consider facial expressions, body cues, and tone of voice; (2) the interaction process was sometimes unnatural; (3) AI chatbots sometimes misunderstood replies from the users; and (4) the content provided by AI chatbots was irrelevant or not interactive enough. Klos et al. (2021) proposed two more limitations of AI chatbots: (5) digital interventions may have limited capacity to capture the motivation and attention of users, and (6) easy access may result in a lower level of commitment to the use of AI. Finally, the requirements for devices equipped with AI may limit the participant pool toward urban areas and higher education levels, introducing bias and potentially reducing the generalizability of AI deployment (Chen et al., 2023; Kleinau et al., 2024). The above limitations suggest new directions for future improvement in the design and functions of AI-assisted interventions.

Several studies raised the concern that the application of AI-assisted intervention was sometimes challenging. Dimeff et al. (2021) reported that successful implementation depended on the willingness of the staff involved to incorporate AI into the workflow. Klos et al. (2021) mentioned the difficulty of accurate translation when applying an established database and algorithm of an AI chatbot to another language. Schillings et al. (2023) proposed that risks such as safety, data privacy, biases, limited empathy, and potential hallucinations in comparison to human interactions require in-depth discussion. All of these challenges may be reduced through greater popularization of AI, supported by evidence-based research, experience in database expansion, technological advancements, and more robust regulation.

In addition to the ethical considerations aligned with the diagnosis and monitoring domains, certain issues discussed in the studies on AI-assisted interventions were particularly important for the treatment of suicidal individuals in the emergency department. Dimeff et al. (2021) reported several ethical practices: (1) an advisory group of people with lived experience with suicide should be involved in developing the use of the AI model, (2) interventions should be drawn upon well-established and evidence-based practice for suicide prevention, (3) a timed protocol stimulation test should be conducted, (4) all procedures should be approved by a board review, and (5) external monitoring should be provided by an independent board of recognized suicide experts. Both Fulmer et al. (2018) and Klos et al. (2021) agreed that (6) crisis support should be provided if users express suicidal ideation, while (7) users should be encouraged to end the chat and reach out for professional help.

This systematic review highlighted the potential of AI in the diagnosis, monitoring, and intervention of mental health disorders. The studies reviewed demonstrated that machine learning algorithms can accurately detect and predict mental health conditions using various predictors, including demographic information, socioeconomic data, clinical history, psychometric data, medical scans, biomarkers, and semantic content. The review also indicated that AI can effectively monitor treatment response and predict the ongoing prognosis of mental health disorders. The studies reviewed in the intervention domain showed that AI-assisted interventions, in the form of chatbots, had the potential to be an effective alternative to traditional in-person interventions and psychoeducation eBooks. The use of AI for intervention assistance in the medical field holds immense promise and warrants further in-depth exploration and research.

The findings of this review can inform AI developers and healthcare practitioners about the development and the choice of AI-based tools and interventions, which can improve the accuracy of mental health diagnosis, treatment, and outcomes. Future directions should focus on developing more robust and diverse datasets and improving the interpretability and transparency of AI models to facilitate their integration into clinical practice.

Two important applications of AI that fall outside the inclusion criteria were discovered during the study selection process of this systematic review. It is important to acknowledge that studies utilizing AI to predict improvements in mental health or symptom remission prior to treatment initiation may still be of significant value for future research. If the accuracy and reliability of these predictions are high, they could serve as useful tools to assist in treatment decision-making. Second, machine learning was adopted in predicting treatment outcomes to facilitate the choice of treatment modality (Delgadillo et al., 2022; Kleinerman et al., 2021) or frequency (Bruijniks et al., 2022). For instance, Kleinerman et al. (2021) found that AI was effective in predicting the treatment outcome prior to treatment initiation and in promoting personalized decision-making. Up to 23% of the participants with depressive symptoms achieved remission earlier without multiple treatment attempts than those in random treatment allocation. It was an impactful study that supported the use of AI in treatment recommendations for better treatment allocation and higher efficiency of treatments. AI was found to have a broader application than the focus of our systematic review, as defined by the inclusion criteria. General limits of AI Common issues observed among included studies were insufficient sample sizes and a lack of diversity in datasets. These limitations lead to imbalanced results and fixed features that compromise model performance. Insufficient diversity can introduce bias given the specific (i.e., limited representation or homogeneous) populations from which the data is drawn while missing data often results in incompleteness, inconsistency, or inaccuracy (Noorbakhsh-Sabet et al., 2019). Such challenges are compounded by noisy and high-dimensional data, making accurate predictions difficult (Noorbakhsh-Sabet et al., 2019). Predictive models also suffer from low input data quality, inadequately representing diverse populations, which hinders their effectiveness (Tejavibulya et al., 2022). Additionally, deep learning models, although capable of reducing dimensionality, are prone to overfitting in contexts with limited training samples, further limiting their predictive capabilities (Noorbakhsh-Sabet et al., 2019). Recognizing and addressing these issues are crucial for optimizing the clinical utility of AI in mental health. Second, the inclusion of singular, excessive, or incomplete variables, as well as the presence of confounding variables, may introduce bias in the analysis. Both the outcome and predictor variables often share common methods, necessitating a strategy to minimize redundancy (Chahar et al., 2021). AI models require transparency and articulation to manage complex interactions (Jha et al., 2021). Since mental health variables exhibit intricate dependencies with potential confounders, it is essential to use data-driven structural learning of Bayesian networks to extend association analyses (Jha et al., 2021). This approach can offer advantages over black-box machine learning and traditional statistical methods by enabling the discovery and modeling of confounding factors transparently (Jha et al., 2021). Standard statistical methods struggle to analyze interactions among numerous variables, whereas structured learning can effectively identify mediation, confounding, and intercausal effects (Jha et al., 2021). Confounding bias is a notable concern. Confounding arises when a variable influences both the exposure and the outcome, generating misleading associations (Prosperi et al., 2020). Observational data, when adjusted for measured confounding – such as through propensity score matching – can help mimic randomized treatment assignment, particularly when using detailed electronic medical records (Prosperi et al., 2020).

Third, some studies lacked effective external validation, which could impact the reliability and generalizability of their findings. External validation in AI mental health research is still rare (Tornero-Costa et al., 2023). Designing appropriate trials for AI applications is challenging due to funding and resource constraints (Tornero-Costa et al., 2023). As a result, retrospective data are often used, raising concerns about its suitability for AI development (Tornero-Costa et al., 2023). Furthermore, some authors may overlook the need for a robust preprocessing pipeline (Tornero-Costa et al., 2023). Consequently, while acknowledging poor model performance, authors often suggest trial-based improvements instead of addressing statistical biases in model development, which could save time and costs (Tornero-Costa et al., 2023). Therefore, before deploying pretrained models, rigorous external validation is necessary to ensure generalizability, which involves testing with independent samples (He et al., 2024). A model should demonstrate excellent generalizability before being considered for commercial use (He et al., 2024). Fourth, balancing different performance metrics poses a challenge in evaluating the effectiveness of AI models consistently. Finally, issues such as the opacity of AI, potential bias or exaggerated predictions, cross-cultural differences, resource constraints, ethical considerations, and technical limitations make the seamless translation of AI findings into real-world applications challenging.

### Real-world applications and future directions

While AI still faces numerous limitations in diagnosis, monitoring, and intervention, it holds vast potential in the healthcare sector, particularly in mental health. AI applied in mental health has more potential than in other healthcare modalities because it allows for a more objective redefinition of psychiatric illnesses, surpassing traditional diagnostic frameworks like the DSM-5 (Alhuwaydi, 2024). Additionally, through advanced techniques such as multimodal emotion recognition and machine learning, AI can facilitate early diagnosis and personalized intervention strategies that adapt to individual patients’ needs, addressing both the obstacles and opportunities in mental healthcare (Alhuwaydi, 2024). To effectively implement AI in clinical settings, researchers and practitioners should focus on developing larger, more diverse datasets and systematic bias detection and correction methods. It is crucial to ensure high data quality and balanced performance metrics to enhance model reliability. Continuous monitoring of AI innovations and maintaining transparency can help to overcome inherent technical constraints (Kiseleva et al., 2022). Additionally, the interactivity of chatbots and the adoption of AI technologies must be prioritized for effective interventions. Maintaining ethical integrity is of paramount importance. Regulatory bodies must guarantee patient privacy, require informed consent, and enhance data security to safeguard ethical standards in AI applications.

Researchers and practitioners should also address the common limits of AI, such as insufficient sample size, lack of diversity, and data quality issues, which can undermine predictive accuracy. Using data-driven structural learning approaches can help to manage complex relationships and minimize confounding biases that may generate misleading results. Prioritizing transparency and articulation in AI models is essential for building trust and ensuring clinical utility. Rigorous external validation is necessary before deploying any pre-trained AI models, as this confirms their generalizability across diverse populations.

### Limitations of the review

This systematic review has some limitations. First, excluding conference papers may have limited the review’s scope, potentially obviating important advancements in AI tools for mental health presented at conferences. Second, the lack of critical analysis of the AI models used in reviewed studies hinders a comprehensive evaluation of their efficacy and reliability in mental health care settings. Third, the exclusion of studies published in languages other than English limits the generalizability of this synthesis as it disregards potentially relevant research findings that may contribute unique insights, methodologies, or outcomes specific to the cultural context of diverse populations.

## Conclusions

This systematic review underscores the significant potential of AI to transform the landscape of mental health diagnosis, monitoring, and intervention. With over half of the studies assessed rated as good in quality, AI methodologies have demonstrated commendable accuracy in detecting and predicting mental health conditions across diverse datasets. Notably, machine learning algorithms showed efficacy in classifying various mental disorders and predicting treatment responses, suggesting a promising pathway for personalized mental health care. However, the review also highlighted critical limitations, including methodological inconsistencies, issues with data quality and diversity, and ethical challenges related to privacy and informed consent. These factors necessitate careful consideration in the development and application of AI tools in clinical practice. The findings inform AI developers and mental health practitioners, advocating for further exploration of data-driven approaches, improved model transparency, and rigorous external validation. Future research should aim to bridge existing gaps and enhance the robustness of AI applications in mental health to ensure they meet the diverse needs of patients effectively and ethically.
